# Distinguishing functional polymorphism from random variation in the sequences of >10,000 *HLA-A*, *-B* and *-C* alleles

**DOI:** 10.1371/journal.pgen.1006862

**Published:** 2017-06-26

**Authors:** James Robinson, Lisbeth A. Guethlein, Nezih Cereb, Soo Young Yang, Paul J. Norman, Steven G. E. Marsh, Peter Parham

**Affiliations:** 1 Anthony Nolan Research Institute, London, United Kingdom; 2 UCL Cancer Institute, University College London, London, United Kingdom; 3 Dept. of Structural Biology & Dept. of Microbiology & Immunology, School of Medicine, Stanford University, Stanford, California, United States of America; 4 Histogenetics, Ossining, New York, United States of America; Children's Hospital of Philadelphia, UNITED STATES

## Abstract

HLA class I glycoproteins contain the functional sites that bind peptide antigens and engage lymphocyte receptors. Recently, clinical application of sequence-based *HLA* typing has uncovered an unprecedented number of novel *HLA* class I alleles. Here we define the nature and extent of the variation in 3,489 *HLA-A*, 4,356 *HLA-B* and 3,111 *HLA-C* alleles. This analysis required development of suites of methods, having general applicability, for comparing and analyzing large numbers of homologous sequences. At least three amino-acid substitutions are present at every position in the polymorphic α_1_ and α_2_ domains of HLA-A, -B and -C. A minority of positions have an incidence >1% for the ‘second’ most frequent nucleotide, comprising 70 positions in *HLA-A*, 85 in *HLA-B* and 54 in *HLA-C*. The majority of these positions have three or four alternative nucleotides. These positions were subject to positive selection and correspond to binding sites for peptides and receptors. Most alleles of *HLA* class I (>80%) are very rare, often identified in one person or family, and they differ by point mutation from older, more common alleles. These alleles with single nucleotide polymorphisms reflect the germ-line mutation rate. Their frequency predicts the human population harbors 8–9 million *HLA* class I variants. The common alleles of human populations comprise 42 core alleles, which represent all selected polymorphism, and recombinants that have assorted this polymorphism.

## Author summary

The HLA complex is a region of the human genome containing immune system genes. Our study concerns those HLA genes that orchestrate defense against viral infections. Distinguishing HLA genes from other human genes is their extensive variation within individuals, families and populations. One advantage of this genetic variation is to increase the depth and breadth of the weaponry used against viruses; another is to impede the spread of infection within families and communities. A drawback to HLA variation is that bone-marrow transplants between donors and patients of different HLA type trigger immune reactions that attack and can kill the patient. For some patients an HLA identical family member can be the donor, but for others an unrelated HLA identical donor is sought. Facilitating these searches are registries, listing millions of possible donors whose HLA types were determined by gene sequencing. During the last ten years, this effort produced exponential growth in the number of HLA variants sequenced. This gave us the unprecedented opportunity to compare more than 10,000 sequences and distinguish aspects of the variation that are important for immune functions, from those that are not. First, however, we needed to develop software that could handle this mass of data.

## Introduction

Present in all jawed vertebrates, the Major Histocompatibility Complex (MHC) is a genomic region that encodes fundamental components of the immune system. Hallmarks of the MHC are highly polymorphic genes that encode diverse MHC class I and II antigen-presenting molecules [1, 2]. The human MHC is called the HLA region and is present on the short arm of chromosome 6 [3]. HLA class I and II glycoproteins have homologous structures and complementary functions in binding peptide antigens and presenting them to lymphocyte receptors [4, 5]. HLA class II is dedicated to adaptive immunity and engagement of the αβ antigen receptors of CD4 T cells [6]. In contrast, HLA class I contributes both to innate immunity, by engaging Natural Killer (NK) cell receptors, and to adaptive immunity, through engagement of the αβ antigen receptors of CD8 T cells [7]. Correlating with these functional differences, polymorphism within the antigen-binding site is restricted to one of the two domains that form the site for HLA class II whereas HLA class I polymorphism is spread throughout the two domains [8, 9]. Consequently, the number of alleles and the differences between them are greater for HLA class I, the subject of our investigation, than HLA class II [10].

Within the *HLA* region, three genes, *HLA-A*, *HLA-B* and *HLA-C*, encode highly polymorphic HLA class I molecules. Sequence variation is concentrated in the α_1_ and α_2_ domains that are encoded by exon 2 and 3, respectively. These two domains contain the binding sites for peptide antigens and lymphocyte receptors [11]. The functional effects of the polymorphism are first to increase the breadth of an individual’s immune response to a pathogen, and second to diversify that response within families and populations. One clinical corollary of *HLA* polymorphism is that numerous diseases are associated with particular *HLA* alleles and haplotypes, and are frequently the strongest genetic associations [7, 12]. Another clinical corollary is that the success of allogeneic transplantation of tissues and organs improves with the extent of HLA match between donor and recipient [13].

HLA class I typing for clinical transplantation was begun in the 1960s using low-resolution serological methods. Nucleotide sequencing of *HLA* class I alleles began in the 1980s and by 1988 had led to establishment of the HLA database as the source for accurate, curated *HLA* sequence data [10, 14–16]. Since that time, improvements in methods [17] have progressively increased the discovery rate of novel alleles. By July 2016 sequences for more than 10,000 *HLA-A*, -*B* and -*C* alleles were deposited in the database. These alleles represent a worldwide sampling of many, but not all, human populations. They provide a unique data set for analysis of HLA class I variation. To analyze this variation, we developed new and general methods for handling and analyzing these large numbers of homologous sequences. Using these tools we examined variation in exons 2 and 3 of *HLA-A*, -*B*, and –*C*, which encode α_1_ and α_2_, with the goal of identifying those aspects of *HLA class I* variation that have most impact on the diversity of human immune function. The methods used here to study exons 2 and 3 of *HLA class I* are directly applicable to polymorphic *HLA class II* genes. They can also be applied to other regions of *HLA* genes, which are known to harbor functionally relevant polymorphism [18–20], when sufficient sequence data become available.

## Results

The α_1_ and α_2_ domains of HLA class I glycoproteins contain the functional sites that bind peptide antigens and engage lymphocyte receptors. These domains are also the site for the extraordinary polymorphism of HLA class I. Clinical sequence-based typing of *HLA-A*, -*B* and -*C*, targets exons 2 and 3 that encode α_1_ and α_2_, respectively. Such typing, of millions of prospective transplant donors, facilitated this analysis of sequence variation in 3,489 *HLA-A*, 4,356 *HLA-B* and 3,111 *HLA-C* alleles (S1 Fig).

### *HLA-A*, -*B* and -*C* are divergent genes with diverse alleles

**Fig 1:**
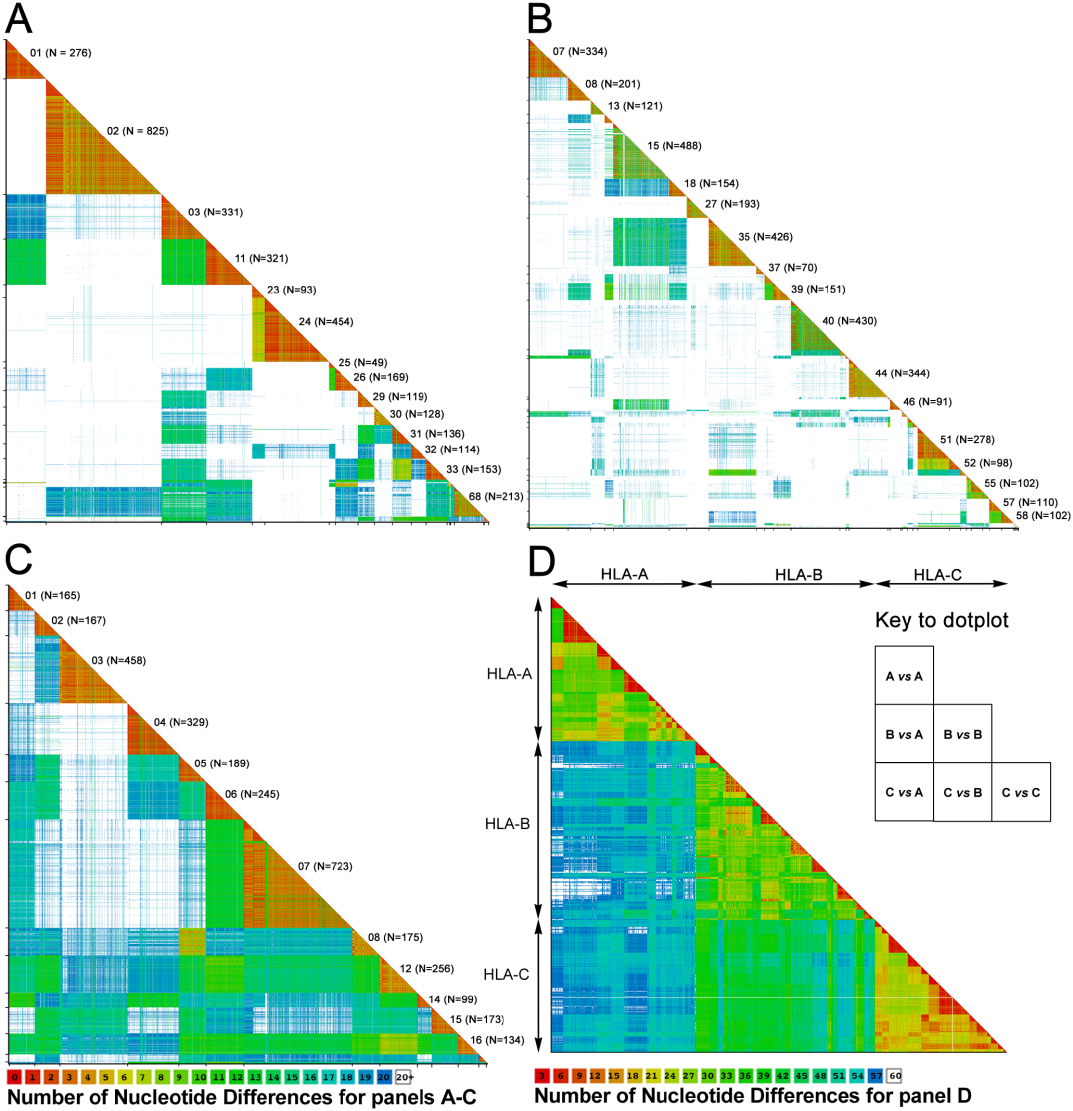
Pairwise comparison defines allele groups with high sequence similarity. The dot plots show the results of pairwise comparison of nucleotide sequences within *HLA-A*, (**Panel A**) *HLA-B* **(Panel B**) *HLA-C* (**Panel C**) and all three combined (**Panel D**). A color scale indicates the number of nucleotide differences in each pair compared with red representing the most closely related alleles. The diagonal labeling in the individual gene plots indicates the allele groups, i.e. 01 in panel A is HLA-A*01, N indicates the number of alleles in each group that were used in the analysis. The diagonal labels do not show the following groups where N is less than 25: A*34, A*36, A*43, A*66, A*69, A*74, A*80, B*42, B*45, B*47, B*49, B*50, B*54, B*59, B*67, B*73, B*78, B*81, B*82, B*83 and C*18.

A general method of multi-sequence dot-plot analysis was developed (see Materials and methods) and used to compare the exon 2 and 3 sequences of *HLA-A*, *HLA-B* and *HLA-C* individually (Fig 1A–1C), and in combination (Fig 1D). The mean intragenic distances of the three genes differ significantly (*p*<1 x 10^−10^, One-Way ANOVA), with *HLA-C* showing the shortest average distance of 16.60 nucleotide differences (3%) compared to *HLA-B*, which has the largest with a mean 27.65 differences between alleles (5%). *HLA-A* is intermediate with 22.82 differences between alleles (4%). The average number of differences between alleles of the same gene is 23.75, whereas the average between alleles of different genes is significantly higher at 51.12 (*p*<1 x 10^−10^, One-Way ANOVA).

The *HLA-A* and *HLA-B* dot plots show well-defined triangular clusters of closely related alleles (Fig 1A and 1B). These clusters correspond to the HLA-A and HLA-B antigens defined by serological typing, the method first used to define HLA class I polymorphisms [21]. Most pairwise differences are greater than 20 nucleotides, producing an extensive white background on which there are well-defined triangles of color. The dot-plot comparison of *HLA-C* alleles also has well-defined clusters corresponding to serological HLA-C types (Fig 1C). However, in contrast to the *HLA-A* and *HLA-B* dot plots, white areas do not dominate because *HLA-C* alleles have diverged to lesser extent than *HLA-A* or *HLA-B* alleles. One likely cause of this difference is that *MHC-A* and *MHC-B* are ten million years older than *MHC-C*, another is that *HLA-C* has distinctive functions in reproduction, which are not shared with *HLA-A* or -*B*. In particular, HLA-C expressed on fetal trophoblast interacts with KIR on maternal uterine NK cells to facilitate placental development [22]. Fig 1D, shows all pairwise comparisons of *HLA-A*, –*B* and -*C* alleles. The color patterns show how *HLA-B* and *HLA-C* are more closely related to each other than either is to *HLA-A*. The median number of differences between sequences of *HLA-B* and *HLA-C* is 42 compared to 55–56 for differences between *HLA-A* and *HLA-B* or *HLA-C* (S9 Fig). These results are consistent with *MHC-C* having originated with duplication of an *MHC-B* allele.

### All positions in the α_1_ and α_2_ domains exhibit variation

Each of the 546 positions in exons 2 and 3 can have five alternative forms, the four different nucleotides and insertion/deletion (indel). The distribution of the variability is shown as histograms in S2 Fig and the numbers per exon for each gene are given in S3 Fig. In summing the data for the three genes, we find only 4.5% of the positions are invariant, whereas 23.2%, 34.3% and 32.2% positions have two, three and four forms, in *HLA-A*, –*B* and –*C*, respectively. All five forms are present at 5.7% of positions. The pattern of variability is similar for *HLA-A*, -*B* and -*C* (S2 Fig). Variation was thus found at almost every position in exons 2 and 3 of these genes.

We performed similar analysis of the amino-acid sequences of the α_1_ and α_2_ domains. The results are displayed as histograms in S4 Fig and summarized in Table 1. The striking result is that, for each of the three genes, there are no positions in the sequences of their protein products that exhibit only one or two amino acids. The number of residues at a given position varies from 3 to 14, with 149 of the 181 positions having between 5 and 9 alternative amino acid residues (Table 1).

**Table 1:** Every position in the α_1_ and α_2_ domains has at least three alternative amino acids.

		Number of positions in domain		
Distinct amino acids	*HLA* gene	α_1_ N = 89	α_2_ N = 92	α_1_ + α_2_ N = 181
**1**	**A**	0	0	0
	**B**	0	0	0
	**C**	0	0	0
**2**	**A**	0	0	0
	**B**	0	0	0
	**C**	0	0	0
**3**	**A**	3	0	3
	**B**	0	0	0
	**C**	3	1	4
**4**	**A**	2	1	3
	**B**	6	1	7
	**C**	7	6	13
**5**	**A**	14	3	17
	**B**	11	2	13
	**C**	14	14	28
**6**	**A**	16	13	29
	**B**	10	12	22
	**C**	23	22	45
**7**	**A**	29	27	56
	**B**	25	13	38
	**C**	19	24	43
**8**	**A**	15	20	35
	**B**	11	18	29
	**C**	12	12	24
**9**	**A**	7	16	23
	**B**	10	14	24
	**C**	6	12	18
**10**	**A**	2	5	7
	**B**	9	12	21
	**C**	3	1	4
**11**	**A**	1	3	4
	**B**	2	9	11
	**C**	1	0	1
**12**	**A**	0	3	3
	**B**	4	5	9
	**C**	1	0	1
**13**	**A**	0	1	1
	**B**	1	3	4
	**C**	0	0	0
**14**	**A**	0	0	0
	**B**	0	3	3
	**C**	0	0	0

### Polymorphic positions comprise a minority of the variable positions

**Fig 2:**
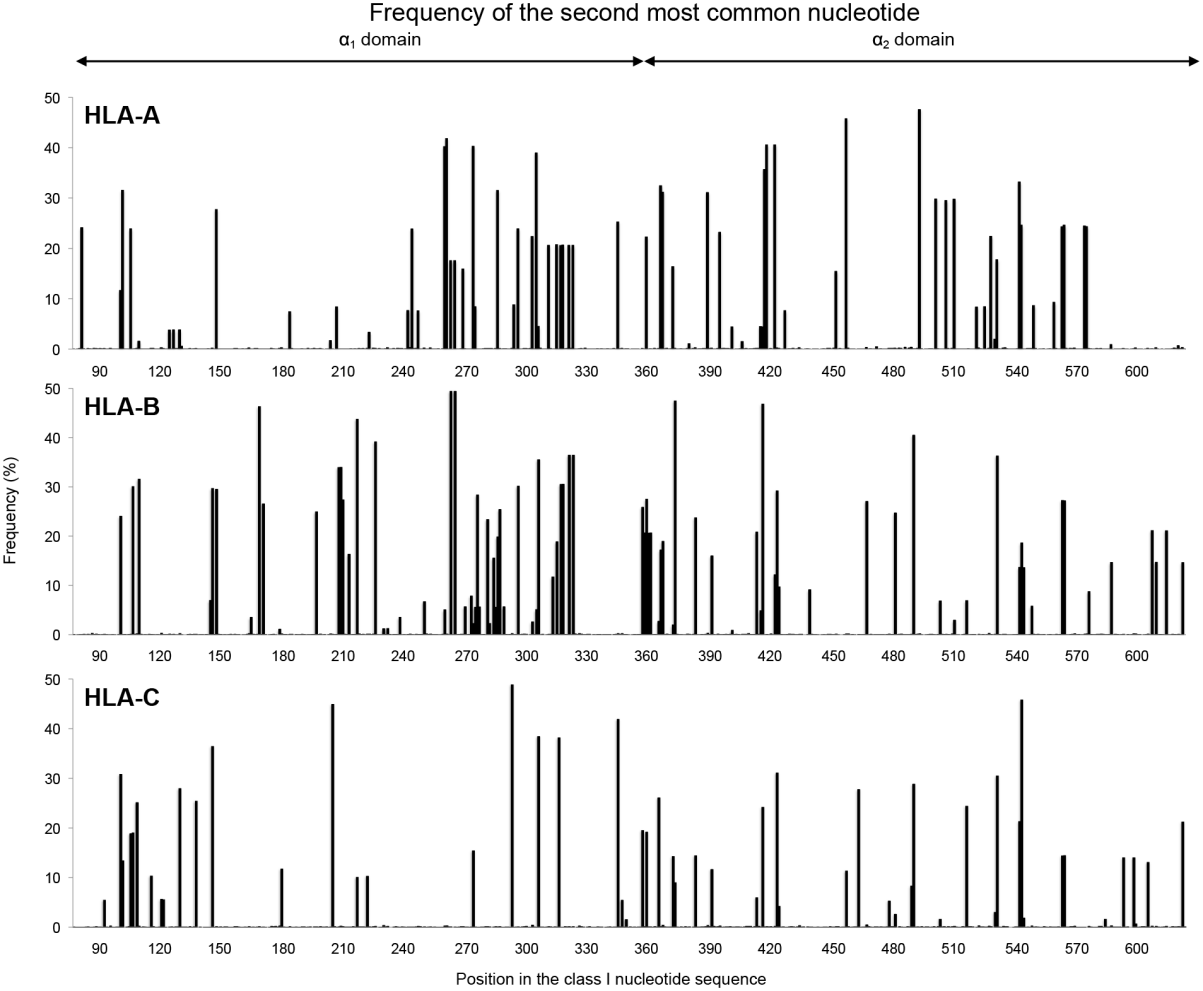
Frequencies of the second most common nucleotide at positions in exons 2 and 3. The histograms show the frequency for *HLA-A* (top) –*B* (center) and –*C* (bottom) of the second most common nucleotide at each position in exons 2 and 3.

To distinguish positions having a balanced polymorphism between two or more nucleotides, from positions dominated by one nucleotide, we determined the incidence (in the dataset of allelic sequences) for the second-most common nucleotide at each position in the exon 2 and 3 sequence (Fig 2). Positions where the incidence of the second nucleotide exceeded 1% were considered polymorphic, whereas positions with lower incidence were considered to exhibit rare variation. The second nucleotide occurs in more than 1% of the alleles for 70 positions in HLA-A, 85 in HLA-B and 54 in HLA-C (S5 Fig). These comprise a minority of positions in the 546 bp sequence of exon 2 and 3, demonstrating that the variation observed at most positions in exons 2 and 3 (S2 Fig) is due to the contribution of nucleotide substitutions that are present in one or a few alleles.

**Fig 3:**
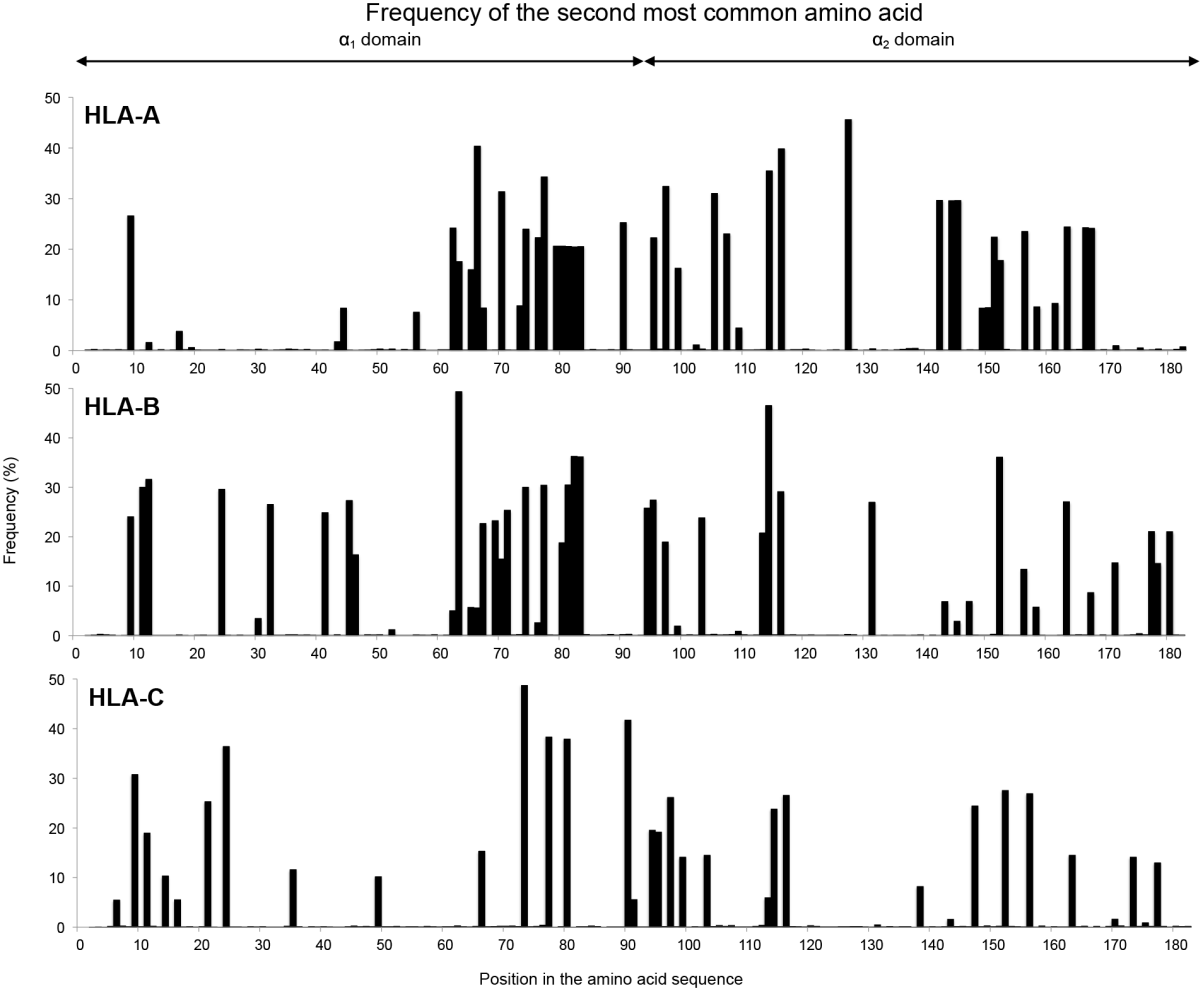
Distribution of polymorphic positions in the α_1_ and α_2_ domains. The figure shows the frequency of the second most common amino acid at positions in the α_1_ and α_2_ domains of HLA-A (top), -B (center) and –C (bottom) allotypes where it has a frequency >1%. Position numbering is based on the mature protein.

**Fig 4:**
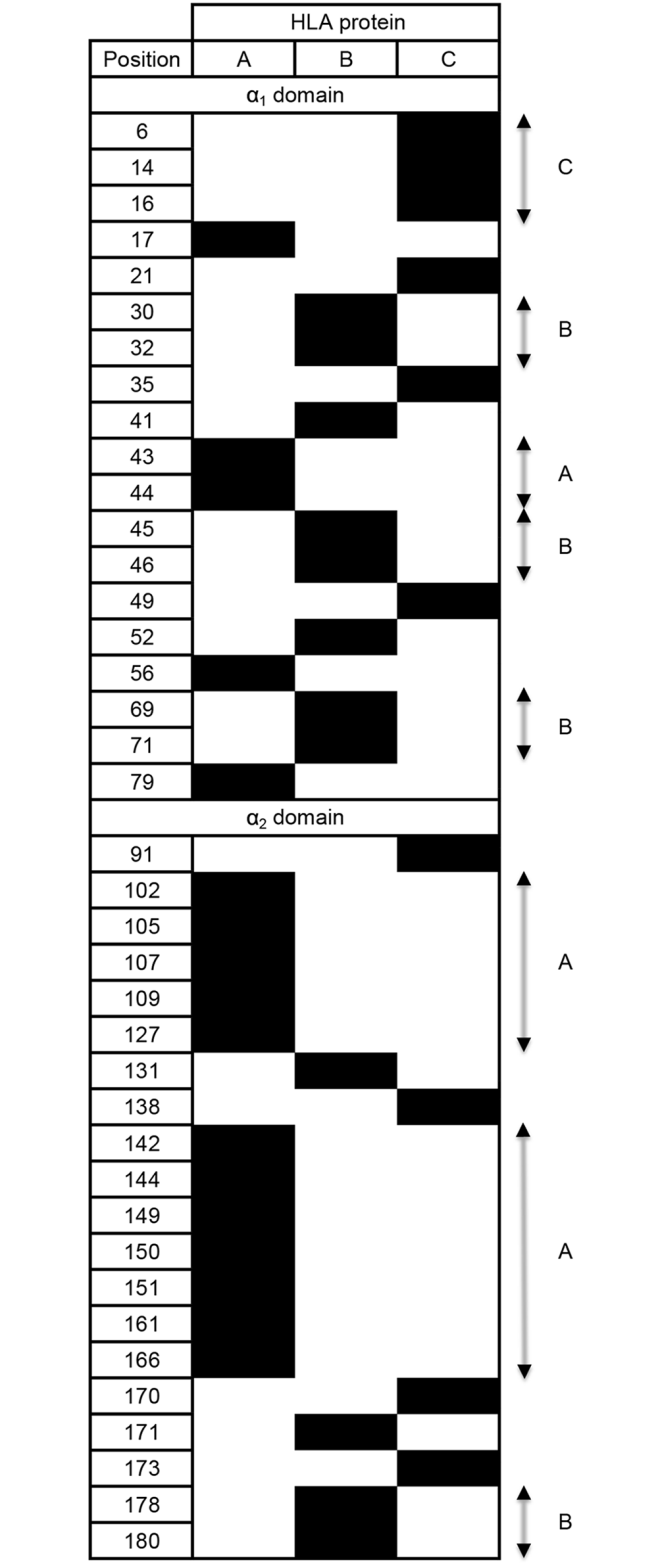
Gene-specific positions of polymorphism. Boxes shaded black denote polymorphic positions (where the second most common amino acid has an incidence >1%) that are only polymorphic in one of the three HLA class I proteins.

Analyzing the incidence of the second most common amino acid residue showed that all 181 positions in the α_1_ and α_2_ domains of HLA-A, -B and -C exhibit some variation. Of these positions, however, only 45 in HLA-A, 46 in HLA-B and 32 in HLA-C have a second amino acid incidence of >1% and are thus considered polymorphic (Fig 3, S6 Fig). Twelve of these positions are shared by HLA-A, -B and -C: four in α_1_ (residues 9, 66, 77 and 80) and eight in α_2_ (95, 97, 99, 114, 116, 152, 156 and 163). Larger numbers of polymorphic positions are shared by two of the three HLA class I: 26 by HLA-A and -B, 20 by HLA-B and -C, and 14 by HLA-A and -C. On the other hand, 17 polymorphic positions are unique to HLA-A, 12 to HLA-B and 10 to HLA-C. These 39 positions impart considerable gene-specific character to the polymorphism (Fig 4). This reflects functional specialization of the three HLA class I.

For polymorphic positions with a second nucleotide incidence of >1%, the mean number of different nucleotides is 3.8 for *HLA-A*, 3.7 for *HLA-B* and 3.6 for *HLA-C*. The values are higher than the mean differences for all other variable positions: 3.1 for *HLA-A* and *HLA-B* and *2*.*9* for *HLA-C*. The polymorphic positions have a significantly increased incidence of three or more nucleotides at each position (91%) when compared to the other positions in the dataset (73%) (Chi squared test, *p* = 2.08 x 10^−6^). Additionally there are polymorphic positions with three or more nucleotides with an incidence of >1%. There are nine positions in *HLA-A*, 14 in *HLA-B* and nine in *HLA-C* having three nucleotides with an incidence >1%. With four nucleotides at an incidence >1% are position 527 (codon 152) in *HLA-A*, positions 206 (codon 45), 272 (codon 67) and 362 (codon 97) in *HLA-B*, and position 368 (codon 99) in HLA-C. These results suggest that variation arising at these sites is more likely to be retained in the population. This is consistent with the sequence variation at such sites serving to diversify the functional interactions of HLA class I with peptide antigens and lymphocyte receptors.

### Polymorphic positions are functional sites subject to positive selection

Crystallographic analyses have identified 70 residues in α_1_ and α_2_ domains of HLA class I that are involved in binding peptide antigens and/or lymphocyte receptors [11, 23–27]. These functionally defined residues overlap considerably with the set of polymorphic residues defined by the incidence of the second nucleotide. Thus, 35 of 45 polymorphic HLA-A positions, 32 of 46 polymorphic HLA-B positions and 19 of 33 polymorphic HLA-C positions are functionally important sites. This correlation of function with polymorphism is highly significant for HLA-A (*p* = 6.52 x 10^−7^) and HLA-B (*p* = 1.18 x 10^−6^), but less so for HLA-C (*p* = 0.0124) (2*x*2 Fisher’s Exact test). The difference is consistent with highly polymorphic HLA-A and -B molecules interacting mainly with highly diverse αβ CD8 T cell receptors, and less polymorphic HLA-C molecules interacting mainly with the less diverse killer cell immunoglobulin–like receptors (KIR) of NK cells.

The striking correlation between immunological function and genetic polymorphism was further investigated by testing the polymorphic sites for evidence of positive selection. Our null hypothesis was that polymorphic sites are not subject to positive selection. If correct there would be no bias in the rates of synonymous and non-synonymous nucleotide substitutions, as measured by the parameters dS and dN. For each test performed, the probability for rejecting the null hypothesis of neutral variation (dN = dS) is shown in Table 2. Values of P<0.05, following a Bonferroni correction and bootstrapping of 1,000 replicates, were considered significant at the 5% level and are highlighted.

**Table 2:** Patterns of selection vary between HLA-A, -B, and -C.

Testing the hypothesis there has been no positive selection on *HLA-A*, -*B* or -*C*							
		Exons 2 and 3 (α_1_+α_2_)		Exon 2 (α_1_)		Exon 3 (α_2_)	
*HLA*	Codons under test	dN-dS	Probability (p)	dN-dS	Probability (p)	dN-dS	Probability (p)
*A*	All codons	1.510	0.8400	0.433	1.0000	1.652	0.6120
	Binding site codons	3.576	0.0031	1.359	1.0000	3.342	0.0067
	Not binding site codons	-1.780	1.0000	-1.433	1.0000	-1.126	1.0000
	2nd Nucleotide > 1%	4.979	0.0001	3.135	0.0130	4.517	0.0008
	2nd Nucleotide not > 1%	-2.661	1.0000	-1.940	1.0000	-1.638	1.0000
	Gene-specific codons	0.585	1.0000	-0.522	1.0000	1.595	0.6794
	Not gene-specific codons	1.150	1.0000	0.272	1.0000	0.251	1.0000
*B*	All codons	0.408	1.0000	0.310	1.0000	0.131	1.0000
	Binding site codons	2.889	0.0275	1.719	0.5280	2.362	0.1187
	Not binding site codons	-1.730	1.0000	-1.190	1.0000	-1.200	1.0000
	2nd Nucleotide > 1%	4.552	0.0001	3.467	0.0044	2.875	0.0286
	2nd Nucleotide not > 1%	-3.060	1.0000	-2.360	1.0000	-1.860	1.0000
	Gene-specific codons	2.444	0.0960	2.220	0.1800	2.358	0.1300
	Not gene-specific codons	-0.041	1.0000	-0.340	1.0000	0.270	1.0000
*C*	All codons	-0.303	1.0000	0.207	1.0000	-0.628	1.0000
	Binding site codons	2.576	0.0720	1.972	0.3000	1.674	0.5760
	Not binding site codons	-1.251	1.0000	-1.280	1.0000	-1.227	1.0000
	2nd Nucleotide > 1%	2.203	0.1800	2.161	0.2400	0.768	1.0000
	2nd Nucleotide not > 1%	-2.780	1.0000	-1.773	1.0000	-2.039	1.0000
	Gene-specific codons	0.989	1.0000	3.653	0.0023	-0.157	1.0000
	Not gene-specific codons	-0.170	1.0000	-0.265	1.0000	-0.023	1.0000

Table 2 shows the results of dN-dS analysis testing for positive selection. For all tests, the probability of rejecting the hypothesis of neutral variation (dN = dS) is shown. P-values of less than 0.05, following a correction for multiple-testing, are considered significant at the 5% level and are highlighted in red. Corrected P-values of 0.0001 are displayed for those values output by MEGA as 0.0000 (indicating a p>0.0001). Corrected P-values exceeding 1.0000, are shown as 1.0000.

We first compared the 70 codons encoding functionally critical α_1_ and α_2_ domain residues (Binding site codons in Table 2), as defined previously [11], to the other 112 codons of exons 2 and 3. For the 70 functional positions, the dN-dS values all point in the direction of positive selection (3.58 for HLA-A, 2.89 for HLA-B and 2.58 for HLA-C) and are statistically significant for HLA-A (*p* = 0.0031) and HLA-B (*p* = 0.0275) but not for HLA-C (*p* = 0.0720) (statistical significance is achieved at p<0.05, after application of Bonferroni correction to the tests on a per gene basis). In contrast, the 112 other positions (Not binding site codons) have negative dN-dS values consistent with the null hypothesis: -1.78 for HLA-A (*p* = 1.0), -1.73 for HLA-B (*p* = 1.0) and -1.25 for HLA-C (*p* = 1.0). These results argue strongly against positive selection at the other positions.

Having validated the selection analysis on functional sites, we compared the polymorphic codons, as defined by having at least one nucleotide position where the incidence of the second nucleotide >1%, with the remaining codons of exons 2 and 3. For the polymorphic codons the dN-dS values pointed clearly in the direction of positive selection and were statistically significant: 4.98 for HLA-A (*p* = 0.0001), 4.55 for HLA-B (*p* = 0.0001) but not for HLA-C (2.20, *p* = 0.1800). In contrast, the values for the codons where the second nucleotide was present at less than 1% were all decidedly negative: -2.66 for HLA-A (*p* = 1.0), -3.06 for HLA-B (*p* = 1.0) and -2.78 for HLA-C (*p* = 1.0). These data strongly support positive selection at the polymorphic positions.

Independent analysis of the α_1_ and α_2_ domains (Table 2) shows that dN-dS for HLA-A is higher in α_2_ for both binding sites and polymorphic positions (3.342, p = 0.0067; 4.517 p = 0.0008) than α_1_ where selection is detected only for polymorphic positions (1.359 p = 1.0000; 3.135 p = 0.0130) which represent a subset of the functionally important residues. For HLA-B selection was detected for the polymorphic positions in both α_1_ (3.467, p = 0.0044) and α_2_ (2.875, p = 0.0286) and for complete set of binding site codons (2.889, p = 0.0275) but not the individual domains. The HLA-C sequences show no significant selection differences between the α_1_ and α_2_ domains, with neither the functional nor polymorphic positions showing significant positive selection.

**Fig 5:**
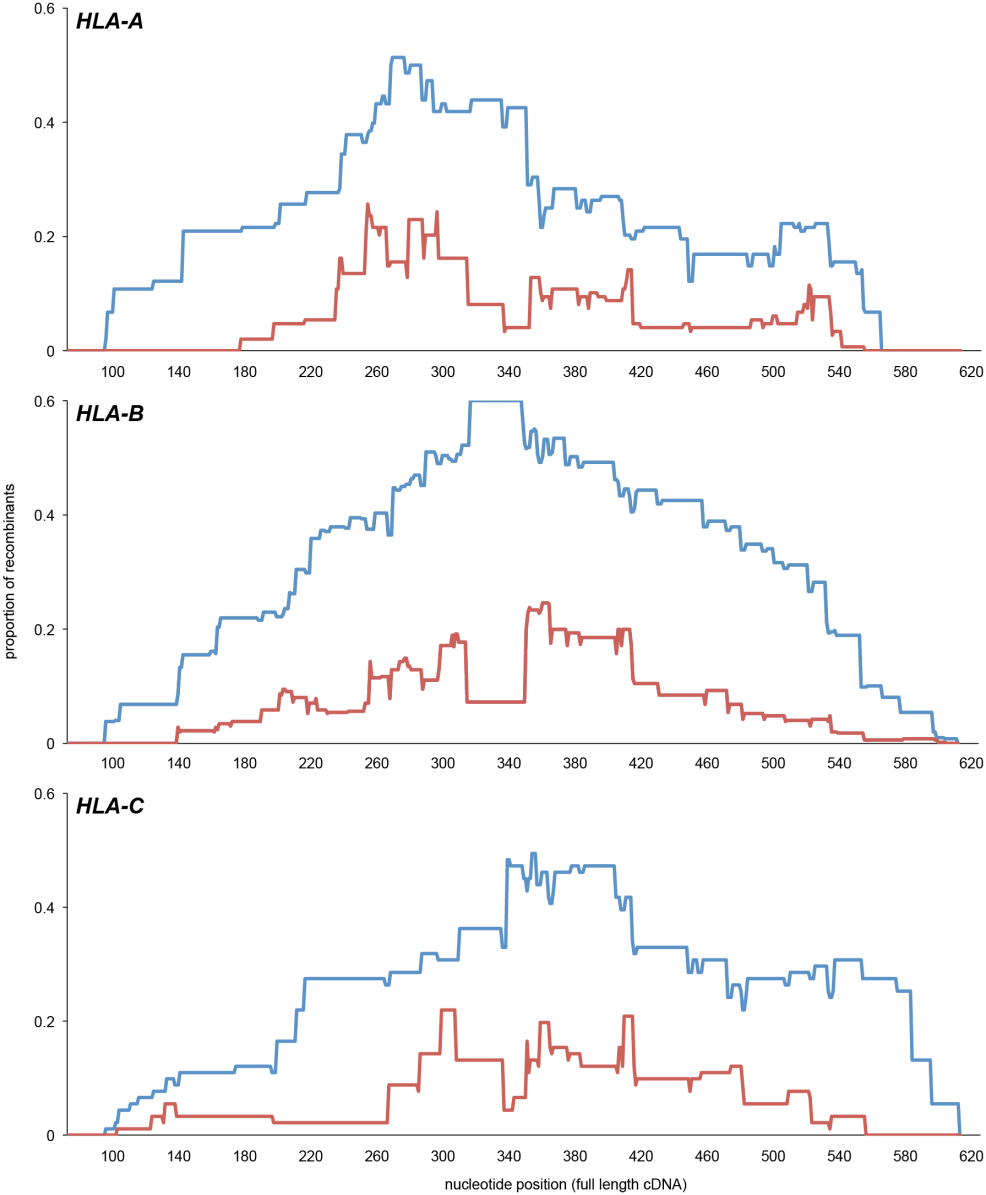
Gene conversion plots. The figure provides a graphical representation of the output from the algorithm used to identify recombinant regions and plots the frequency with which each position is likely to part of a recombinant region. The two lines represent the minimum (red) and maximum (blue) potential regions likely to have been subject to recombination. The region with the greatest proportion of recombinants in the *HLA-B* graph, between positions 300 and 325, maps to the region encoding the Bw4 motif, which is known to have recombined between different *HLA-B* allele groups.

Assessment of selection at gene-specific positions of polymorphism (Fig 5) showed there has been positive selection only for HLA-C specific polymorphisms and those are limited to one of the two domains. The α_1_ domain has been subject to strong positive selection (dN-dS = 3.65, *p* = 0.0023), but that is not the case for HLA-C specific sites of α_2_ (dN-dS = -0.16, *p* = 1.00). The gene-specific sites of HLA-A and HLA-B show no evidence for significant positive selection.

### Distinguishing SNP and recombinant HLA class I alleles

Previous analysis of HLA class I variation, studied small numbers of alleles and relied on visual inspection to discern the relationships between them [5]. To analyze the current dataset of 10,956 *HLA* class I sequences, we developed the Sq2 algorithm (see Materials and methods), which provides a quicker, more objective and largely automated approach. In two separate phases of analysis, Sq2 divided the alleles into three categories.

In the first phase, Sq2 identified all SNP alleles, which constitute ~85% of the dataset. These are alleles of more recent origin that differ from an older allele by just one nucleotide substitution. After identifying and removing the SNP alleles, the reduced database of 1,555 alleles was subjected to the second phase of analysis. This identified all alleles that are recombinants of other alleles. To do this, Sq2 identified motifs of several substitutions that are present in multiple allelic backgrounds as a consequence of recombination (Fig 5). The iconic example is the *Bw4* motif. Present in codons 76–83 of one third of *HLA-B* alleles, *Bw4* defines the ligand recognized by a major NK cell receptor, KIR3DL1 [28, 29]. As well as being present in 12 of the 33 *HLA-B* allele families, *Bw4* was transferred by a gene conversion from *HLA-B* to *HLA-A*, where it spread by recombination to four *HLA-A* allele families [30]. By comparing the distribution of such motifs among alleles, Sq2 identified pairs of alleles differing only by presence or absence of a particular motif.

In this way 1,171 recombinants were identified. Of these 1,092 were formed by recombination between alleles of the same gene (intragenic recombinants), and 79 were recombinants formed by recombination between alleles of different genes (intergenic recombinants). Of the latter, 16 are products of single recombination (crossover) and 63 (*10 HLA-A*, 37 *HLA-B*, and *16 HLA-C*) are products of double recombination (conversion). *HLA-B* is clearly seen as the more frequent beneficiary of recombination (Table 3).

**Table 3:** HLA class I alleles can be divided into three categories.

Categories of HLA class I alleles									
HLA gene	Number of alleles							SNP alleles	
	Total	Distinct Sequences	Core alleles	Recombinants		Other mechanisms	SNP alleles	Fraction of total sequences (%)	
				Intragenic	Intergenic			Total	Distinct
A	3,489	3,170	11	226	10	127	2,796	80.1	88.2
B	4,356	4,072	17	728	47	92	3,188	73.2	78.3
C	3,111	2,872	14	138	22	123	2,575	82.8	89.7
All	10,956	10,114	42	1,092	79	342	8,559	78.1	84.6

Each allele can be assigned as a core allele, a SNP allele or a recombinant allele. The number of alleles in each of these categories is shown. Total refers to all sequences covering exons 2 and 3 at each gene Distinct Sequences refers to the unique sequences over exons 2 and 3. Recombinants refers to alleles formed by either Intra or Inter-genic mechanisms. Other mechanisms counts the alleles that cannot be assigned to a particular mechanisms.

Among intragenic recombinants, double recombinants (N = 735) outnumber single recombinants (N = 357) by a factor of two. It is likely that some alleles assigned as single recombinants are actually double recombinants, for which the second recombination is not in exon 2 or 3 but in a flanking intron, for which we had no sequence. Both forms of recombinant are more prevalent at *HLA-B* (N = 728) than either *HLA-A* (N = 226) or *HLA-C* (N = 138). The frequency of double recombination for *HLA-B* is similar in exons 2 and 3, whereas it is greater in exon 2 of *HLA-A* and in exon 3 of *HLA-C*. A similar hierarchy is observed for the single recombinants.

### A set of core alleles represents all elements of HLA class I polymorphism

**Fig 6:**
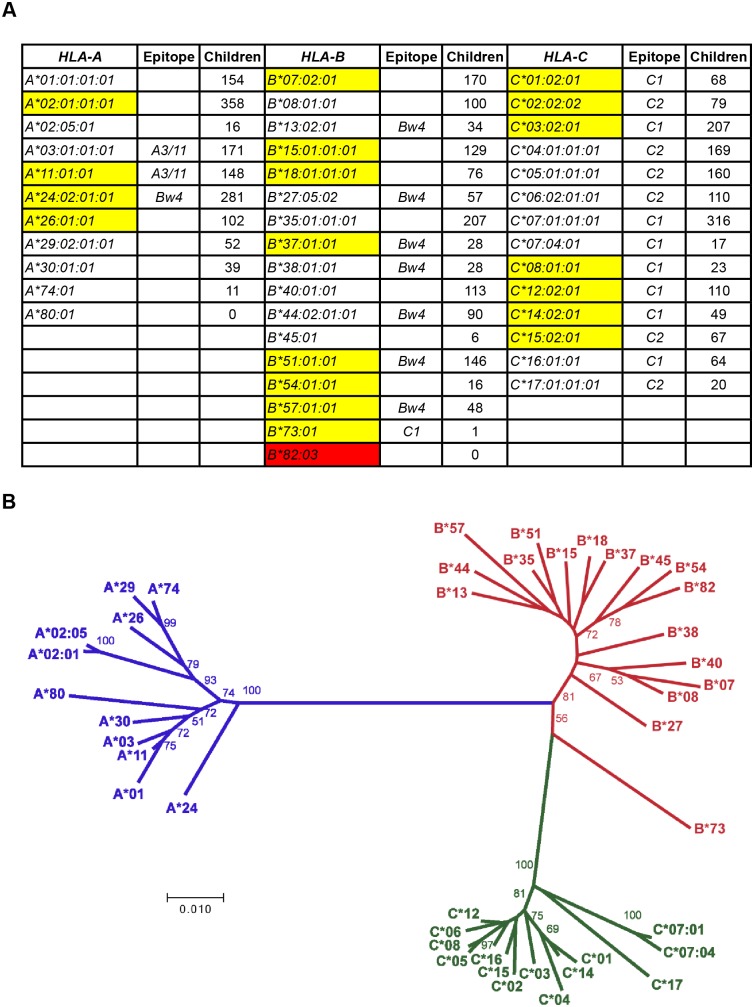
Core *HLA-A*, -*B* and –*C* alleles. **A** Starting with the 42 core alleles (11 *HLA-A*, 17 *HLA-B* and 14 *HLA-C*) it is possible to derive all the other *HLA-A*, -*B* and –*C* alleles by events of recombination and point mutation. This is the minimum number of alleles by which this can be achieved. The core alleles are not meant to represent any particular human population, either ancient or modern. The yellow shading indicates potential archaic alleles that have been transmitted from Denisovans or Neanderthals. Red shading indicates rare allele that does not have a frequency > 0.001 in more than one reference population. **B** An unrooted phylogenetic tree of the core alleles. Numbers at the nodes indicate bootstrap support. Where a number is absent, support at that node was < 50.

Removal of SNP and recombinant alleles, reduced the database to <1% of its original size. This left *11 HLA-A*, *17 HLA-B* and *14 HLA-C* alleles (Fig 6A). Because these 42 alleles represent all functionally significant variation (polymorphism) in exons 2 and 3 of HLA-A, -B and -C, we call them ‘core’ alleles (Fig 6B). Although they are older in their origins than the SNP alleles and recombinant alleles, they are unlikely to represent, or reflect, any particular human population, either ancient or modern.

Core alleles vary widely in their contribution to the total set of alleles (Fig 6A), in their geographical distribution (S7 Fig) and in their abundance in the modern human population. A substantial proportion of the core alleles, 5 *HLA-A*, 8 *HLA-B* and 6 *HLA-C*, are likely derived from archaic humans (Fig 6A) [31]. A dot plot analysis of the core alleles (S8 Fig) has similar substructure to that of the complete set of alleles (Fig 1D) and for each gene the mean pairwise differences for core alleles and all alleles is remarkably similar (S9 Fig). Analysis of selection on the polymorphic and functional sites of core *HLA-A*, -*B* and -C alleles (Table 4) gives comparable results to those obtained for the full sets of alleles (Table 2) for *HLA-A*. For *HLA-B* and -*C* the results are comparable when looking at the full-length sequence, but some differences are seen for the individual domains. This could, however, be due to the small number of sequences analyzed.

**Table 4:** Patterns of selection vary between core alleles of HLA-A, -B, and -C.

Testing the hypothesis there has been no positive selection on *HLA-A*, -*B* or -*C core alleles*							
		Exons 2 and 3 (α_1_+α_2_)		Exon 2 (α_1_)		Exon 3 (α_2_)	
*HLA*	Codons under test	dN-dS	Probability (*p*)	dN-dS	Probability (*p*)	dN-dS	Probability (*p*)
*A*	All codons	1.112	1.0000	-0.170	1.0000	1.825	0.4200
	Binding site codons	2.421	0.0960	0.470	1.0000	2.821	0.0360
	Not binding site codons	-1.639	1.0000	-1.462	1.0000	-0.706	1.0000
	2nd Nucleotide > 1%	6.016	0.0001	3.305	0.0041	5.102	0.0001
	2nd Nucleotide not > 1%	-2.936	1.0000	-2.165	1.0000	-2.129	1.0000
	Gene-specific codons	1.372	1.0000	0.135	1.0000	2.670	0.0480
	Not gene-specific codons	0.368	1.0000	-0.084	1.0000	0.483	1.0000
*B*	All codons	0.250	1.0000	0.240	1.0000	0.041	1.0000
	Binding site codons	2.401	0.1080	1.428	0.9360	1.577	0.7080
	Not binding site codons	-1.993	1.0000	-1.461	1.0000	-1.404	1.0000
	2nd Nucleotide > 1%	4.023	0.0006	3.568	0.0028	1.718	0.5280
	2nd Nucleotide not > 1%	-3.150	1.0000	-2.697	1.0000	-1.609	1.0000
	Gene-specific codons	2.905	0.0262	-0.479	1.0000	3.190	0.0130
	Not gene-specific codons	-0.349	1.0000	-0.468	1.0000	-0.008	1.0000
*C*	All codons	-0.100	1.0000	-0.254	1.0000	0.021	1.0000
	Binding site codons	2.611	0.0600	0.733	1.0000	2.229	0.1680
	Not binding site codons	-2.111	1.0000	-1.803	1.0000	-1.738	1.0000
	2nd Nucleotide > 1%	2.926	0.0246	2.516	0.0840	0.844	1.0000
	2nd Nucleotide not > 1%	-2.821	1.0000	-1.958	1.0000	-2.076	1.0000
	Gene-specific codons	0.609	1.0000	5.519	0.0001	-1.713	1.0000
	Not gene-specific codons	-2.897	1.0000	-0.847	1.0000	0.776	1.0000

Table 4 shows the results of dN-dS analysis testing for positive selection. For all tests, the probability of rejecting the hypothesis of neutral variation (dN = dS) is shown. P-values of less than 0.05, following a correction for multiple-testing, are considered significant at the 5% level and are highlighted in red. Corrected P-values of 0.0001 are displayed for those values output by MEGA as 0.0000 (indicating a p>0.0001). Corrected P-values exceeding 1.0000, are shown as 1.0000.

**Fig 7:**
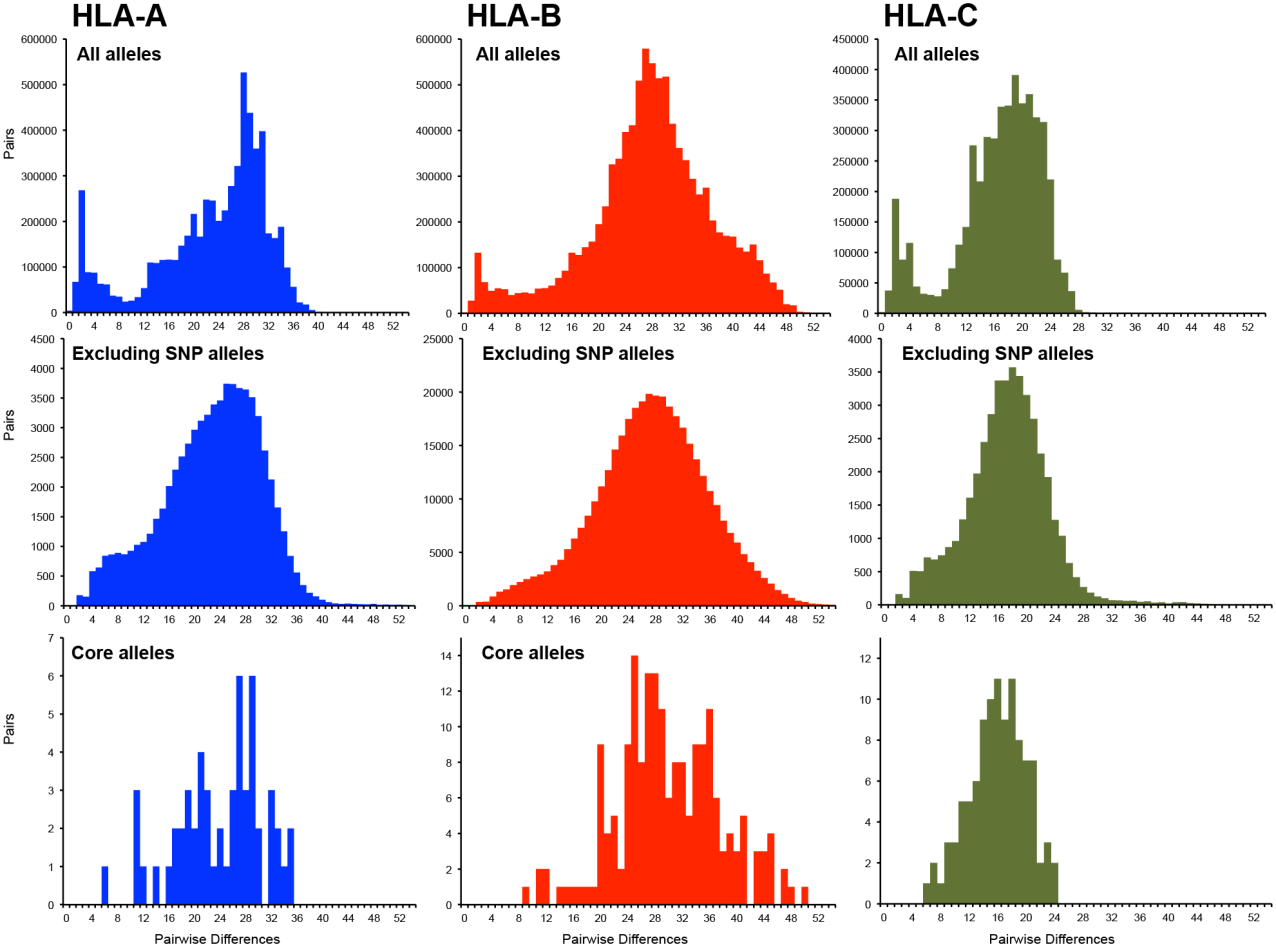
Pairwise distances of alleles form characteristic distributions for *HLA-A*, -*B* and –*C*. Shows the distribution of pairwise differences for all alleles (top row), core alleles and recombinant alleles (center row), and core alleles alone (bottom row) for *HLA-A* (left column), *HLA-B* (center column) and *HLA-C* (right column).

The effects of applying the Sq2 algorithm to the HLA class I data set are seen in histograms constructed from the pairwise differences of nucleotide sequences (Fig 7, top row). For complete sets of *HLA-A*, -*B* and -*C* alleles, the histograms have a characteristic bimodal distribution with one peak at 2 nucleotide differences and a second peak at 20–30 nucleotide differences. The first peak contains the large number of pairwise comparisons between alleles differing by one or two nucleotide substitutions. Pairs differing by one nucleotide substitution usually involve an older, common allele and a rare SNP variant. Pairs differing by two nucleotide substitutions involve two rare SNP variants that differ from the same parental allele by different SNPs.

Taking the SNP alleles out of the analysis, led to loss of the first peak and retention of the second peak (Fig 7, middle row). For *HLA-A* and -*B* the loss is complete, but for *HLA-C* it is not. *HLA-A* gives a bimodal distribution, which differs from that observed in the complete dataset. This is because *HLA-A* comprises a small number of large and divergent allele families. Thus the minor distribution, seen as the shoulder at 4–12 nucleotide differences, comprises the differences between members of the same allele family, whereas the major distribution is formed from the larger differences between members of different allele families. In contrast to *HLA-A*, *HLA-B* comprises a large number of less divergent allele families than *HLA-A*, as well as a few highly divergent alleles with no close family ties. This gives *HLA-B* both a more symmetrical and broader distribution.

Histograms for the pairwise differences between core alleles (Fig 7, bottom row) represent much of the range of difference seen with the larger data sets, with the notable absence of allele pairs differing by small numbers of substitutions. That the *HLA-C* core allele histogram has a distribution with a more coherent shape, than the *HLA-A* and -*B* core histograms, probably reflects the more recent origin of *HLA-C* [4, 32].

### The human population is estimated to have millions of *HLA class I* alleles

Because we have detected variation at all nucleotide positions in exons 2 and 3 of *HLA-A*, -*B* and -*C* (S2 Fig) the maximum number of possible *HLA class I* alleles is 5^546^ (4.3 x 10^381^). This calculation is based on observing all four nucleotides or an indel at each of the 546 positions in the exon 2 and 3 sequence. This number far exceeds the size of the modern human population, which is estimated to be 7.5 billion (http://www.worldometers.info/world-population/). This difference means that the number of variants present in a population is limited only by the size of that population.

To estimate the total number of *HLA-A*, -*B* and -*C* alleles now present in the human population, we first determined the rate at which novel alleles are being identified. In this context, the rate is simply the ratio between the number of individuals typed and the number of new alleles discovered. For each gene, the product of the rate and the population size (7.5 billion) gives an estimate of the total number of alleles. To provide an internally consistent dataset, we analyzed *HLA* typing data from donor cohorts recruited by various transplantation registries, but all typed at the same sequencing center (Histogenetics).

Similar rates, of 1.80, 2.13, and 2.18 x 10^−4^, were observed for the acquisition of novel *HLA-A*, -*B* and –*C* alleles, respectively (Table 5). Using these rates, we estimate there are 2.7 million *HLA-A*, 3.3 million *HLA-B* and 3.2 million *HLA-C* alleles in today’s human population. These estimates are comparable to the 3.5 million alleles per *HLA* gene predicted by Klitz, et al [33], using estimates of effective population size and mutation rates.

**Table 5:** Rates of new allele identification.

*HLA* gene	Number of individuals tested	Number of novel alleles	Frequency of novel alleles
***A***	3,255,436	1,177	1.80 x 10^−4^
***B***	3,260,166	1,422	2.18 x 10^−4^
***C***	3,012,398	1,284	2.13 x 10^−4^

On average, *HLA* typing ~2,000 individuals yielded one novel allele for each of the *HLA* class I genes three genes. Of 6,956 different HLA class I alleles identified, 3,883 were novel alleles detected in only one individual or family.

Our method for estimating the total numbers of *HLA-A*, -*B* and -*C* alleles used a constant rate for the discovery of novel alleles. This assumption was based on the results of two recently published studies [34, 35], which both indicated that the rate of discovery of new alleles is not tapering off over time, even for European populations [34, 35], which have been intensively studied compared to the populations of other continents.

Analyses show that the human population has a small number of common *HLA* class I alleles (68 *HLA-A*, 125 *HLA-B*, 44 *HLA-C*) that are present at appreciable frequency in different populations [36]. In contrast, the overwhelming majority of *HLA* class I alleles are very rare and highly localized in their distribution. Consistent with these properties, each newly sampled cohort or population is expected to harbor a subset of *HLA-A*, -*B* and -*C* alleles that are novel and present in only one or a few individuals. Because of their rarity and population specificity, the relative frequency of novel alleles will not diminish in time as further cohorts of donor are *HLA* typed at high resolution.

## Discussion

We studied sequence variation in exons 2 and 3 that encode the highly polymorphic α_1_ and α_2_ domains of HLA-A, -B and -C. The analysis was restricted to these exons and genes to enable an in depth study of the maximum number of sequences. The tools developed for this analysis can, and should, be extended to study the remaining exons of these genes, which are known to contain functionally relevant polymorphism [18–20], when sufficient data becomes available. These analyses can also be applied to the study of polymorphism in the *HLA* class II genes.

Sequence differences in the α_1_ and α_2_ domains of HLA-A, -B and -C determine the peptide antigens that are bound by an HLA class I allotype, as well as the lymphocyte receptors that can engage the complex of peptide and HLA class I. *HLA-A*, -*B* and -*C* are candidates for being the most polymorphic of human genes [22]. Moreover, their polymorphisms are associated with numerous clinical factors including infectious diseases, autoimmune and inflammatory diseases, pregnancy syndromes and success in the transplantation of allogeneic organs and tissues [7, 37–42].

Transplantation of bone marrow, and other sources of hematopoietic stem cells, is a successful and widely used therapy for leukemias, lymphomas and other malignancies of hematopoietic cells. The preferred donor is an HLA identical sibling, but in the absence of such a donor, the next best choice is an unrelated individual having the same, or very similar, HLA type as the patient. To identify such donors, there exists an international network of donor registries, which has HLA typed more than 30 million potential HCT donors [43]. During the last ten years, less precise methods of HLA typing have been superseded by nucleotide sequencing exons 2 and 3 of the *HLA-A*, –*B* and –*C* genes.

The set of *HLA* class I sequences we studied derive from sequence-based typing of >3 million individuals, as well as earlier studies in which typing at lower levels of resolution identified variants, which were followed up with targeted sequence analysis. The prospective donors of hematopoietic stem cells were recruited to registries in varied countries and continents, but demographically and anthropologically they are not, in the main, well characterized. A total of 10,956 different exon 2 and 3 sequences were analyzed: 3,489 *HLA-A*, 4,356 *HLA-B* and 3,111 *HLA-C* alleles. In our analysis of these three sets of alleles, each sequence was given equal weight, irrespective of its abundance or scarcity in any human population.

At the nucleotide level, we found substitutions at >95% of all positions in each of the three genes. As the exceptions are at different positions in each gene, we predict that substitution at these positions will soon be identified. At the amino-acid level, we found substitutions at every position in the α_1_ and α_2_ domains of HLA-A, -B and –C. A majority of the substitutions, >84%, are in rare alleles, which in many cases have been detected in only one individual or one family. Most of the alleles differ from a common allele by the single substitution that defines them. The obvious interpretation of these data is that these substitutions reflect the germ line mutation rate of the *HLA-A*, –*B* and –*C* genes. Consistent with this thesis, there is no evidence for positive selection at these sites, many of which are, otherwise, highly conserved. The remaining alleles are formed by intragenic or, rarely, intergenic recombination events. From the rate at which new alleles in exons 2 and 3 have been defined by sequence-based typing we estimate there are 2–3 million each of *HLA-A*, -*B* and -*C* alleles in the human population worldwide.

The majority of the variable nucleotide positions are characterized by one dominant and one or more rare nucleotides. However, variation at a smaller number of nucleotide positions, (70, 85 and 54 in *HLA-A*, -*B* and -*C*, respectively) has a very different character. These positions have two, three or four nucleotides at appreciable frequency. They have also been spread by recombination throughout the population of alleles and are thus found in numerous combinations. There is good evidence for positive selection at these sites, which has over time, given them a balanced polymorphism. Supporting this conclusion, numerous immunological studies have correlated substitution at polymorphic sites with modulation of HLA-A, -B and –C function [7, 37, 40, 41, 44–46]. Thus we can divide the alleles into two distinctive groups. Firstly SNP alleles, defined by substitution that confers no functional benefit, but could be detrimental in the context of transplantation. Secondly, functional alleles, with functional benefit conferred by combinations of substitutions at positions with balanced polymorphism.

We further divided the functional alleles into two subgroups: 1,171 recombinant alleles that were derived by recombination from other alleles and 42 core alleles (11 *HLA-A*, 17 *HLA-B* and 14 *HLA-C*) that cannot be derived by simple events of recombination from other alleles. The core alleles, many of which were passed by introgression from archaic to modern humans [31], contain all elements of HLA-A, -B and –C polymorphism present in the modern human population. Although the core alleles are probably older than the SNP alleles and the recombinant alleles, they are very unlikely to represent the *HLA-A*, -*B* and -*C* alleles carried by any particular ancestral human population.

Because polymorphic *MHC class I* and *II* genes have no wild-type, understanding their genetics and biology in any species requires extensive study of populations. For reasons of cost and logistics this has been rarely, if ever, achieved. Many population studies have recruited only small numbers of individuals (therefore, likely missing rare alleles) and until recently have reliably assayed only known alleles. Because the *HLA class I* and *II* genes contribute to so many numerous and diverse aspects of human health and disease [7, 37–42], the *MHC* of the human species is by far the most studied and, by default, provides the model for studies of other placental mammals [4, 32, 47–49]. The capacity to acquire large datasets, of the type we have analyzed and reported here, should enable *HLA* population genetics and disease associations to be studied to increasingly higher definition, resolution and coverage of the world’s human populations.

## Materials and methods

### The HLA class I sequence dataset

The minimum requirement for naming an *HLA class I* allele and depositing it in the IPD-IMGT/HLA Database, is the nucleotide sequence of exons 2 and 3. Because of this requirement, a majority of deposited sequences (~65% of *HLA-A* and -*B* alleles and ~80% of *HLA-C* alleles) consist of only exons 2 and 3, encoding residues 2-182 of the mature HLA class I protein. Thus to maximize the number of alleles analyzed we limited this study to the sequences of exons 2 and 3. Our analysis used all sequences in the IPD-IMGT/HLA database as of July 2016 (Release 3.25.0).

### Methods of sequence comparison and analysis

All analyses used custom written Perl scripts, http://www.perl.org [50], with graphical outputs generated using the Perl::GD modules or R, http://www.r-project.org [51]. Where appropriate, statistical analysis was also completed in R. For F distribution analyses with df1 and df2 exceeding 1,000, R outputs a p-value of 0, these have been reported as *p*<1 x 10^−10^. The set of scripts developed constitutes the Sq2 package. Individual scripts perform different steps of the algorithm. The individual algorithms are listed and described below:

1. *sq2_basealigner*.*pl*—prepares the sequence for alignment, utilizes ClustalW and then formats the alignment for use in further scripts.
2. *sq2_msdotplot*.*pl*—generates the multiple sequence dot plots
3. *sq2_histovariant*.*pl*—is used to measure the diversity at each position and plot the values as a histogram
4. *sq2_identifyf2*.*pl*—is used to identify polymorphic positions, where the second allele is at a set frequency
5. *sq2_removemono*.*pl*—is a perl module used to remove invariant positions from sequence-alignment libraries
6. *sq2_v3*.*pl*—this perl script has options to identify SNP and recombinant variants
7. *sq2_recombiner*.*pl*—this perl script identifies the recombinant regions between groups of sequences.

The scripts are available from the ANHIG Gitlab repository which can be found at; https://github.com/ANHIG.

#### Sequence alignment—*sq2_basealigner*.*pl*

For each of the three genes the combined exon 2 and 3 sequences of all alleles were aligned using the ClustalW alignment tool [52]. The output was processed using *sq2_basealignmer*.*pl*, which removed redundant sequences and recoded insertions and deletions so as to preserve the alignment. Redundant sequences arise because some alleles differ only by non-coding region substitutions, or coding region substitutions outside of exons 2 and 3. The script, *sq2_basealigner*.*pl*, removes all but one of a set of repeated sequences. The retained sequence is that with the lowest number in the allelic series. In recoding an insertion, the sequence of the insertion and the preceding nucleotide were both replaced by the letter ‘I’ for indel. In recoding a deletion, each one of the deleted nucleotides was represented in the sequence by the letter ‘I’.

To shorten computation times and give clarity when viewing the output of analyses, the *sq2_removemono*.*pl* was developed to remove invariant positions from sequence alignments. Depending on the gene, between 7–14 positions in exon 2 and 11–16 positions in exon 3 were removed from the alignments.

#### Dot plot comparison of HLA class I sequences—*sq2_msdotplot*.*pl*

Dot plots have been widely used to compare pairs of sequences [53]. In expanding this concept, we developed a multiple sequence dot plot method (*sq2_msdotplot*.*pl*) that compares any number of sequences in a single plot. Dot plots were made from the exon 2 and 3 sequences of *HLA-A*, *HLA-B* and *HLA-C* alleles, separately, as well as in combination. In these plots, compared sequences are listed along both the x and y axes. Each pairwise comparison is represented by a colored dot on a two dimensional plot. The color of the dot indicates the similarity of the sequences: red indicating identity, proceeding through orange, yellow, green, and blue to white as the similarity decreases. A characteristic diagonal red line is produced by all comparisons of a sequence with itself.

#### Histograms of sequence variation—*sq2_histovariant*.*pl*

The script, *sq2_histovariant*.*pl*, was developed to take aligned *HLA-A*, -*B* and -*C* sequences and generate histograms for the number of different nucleotides, as well as insertion-deletion (I) that occur at each position. Amino-acid sequence variation of HLA-A, -B and -C allotypes was similarly analyzed using *sq2_histovariant*.*pl*.

The initial set of histograms determined which nucleotides occur at each position, but gave no measure of their relative frequencies in the allele population. To provide this measure, we developed the *sq2_identifyf2*.*pl* script that constructs a second set of histograms giving the frequency of the second most common nucleotide at each position.

### Using Sq2 to segregate mutant, recombinant and core *HLA-A, -B* and -*C* alleles

Alleles of an HLA class I gene are of three types: core alleles, recombinant alleles and SNP alleles. Core alleles comprise the set of alleles that cannot be related to each other by single events of recombination or point mutation. Recombinant alleles are the products of one or more recombination events between core alleles. SNP alleles differ from another allele by a single nucleotide polymorphism (SNP). The Sq2 algorithm was developed to assign *HLA class I* alleles to these three categories. In a series of iterative steps, Sq2 first defines the mutant alleles, then the recombinant alleles and lastly the core alleles.

#### Identification of mutant alleles—*sq2_v3*.*pl*

**Fig 8:**
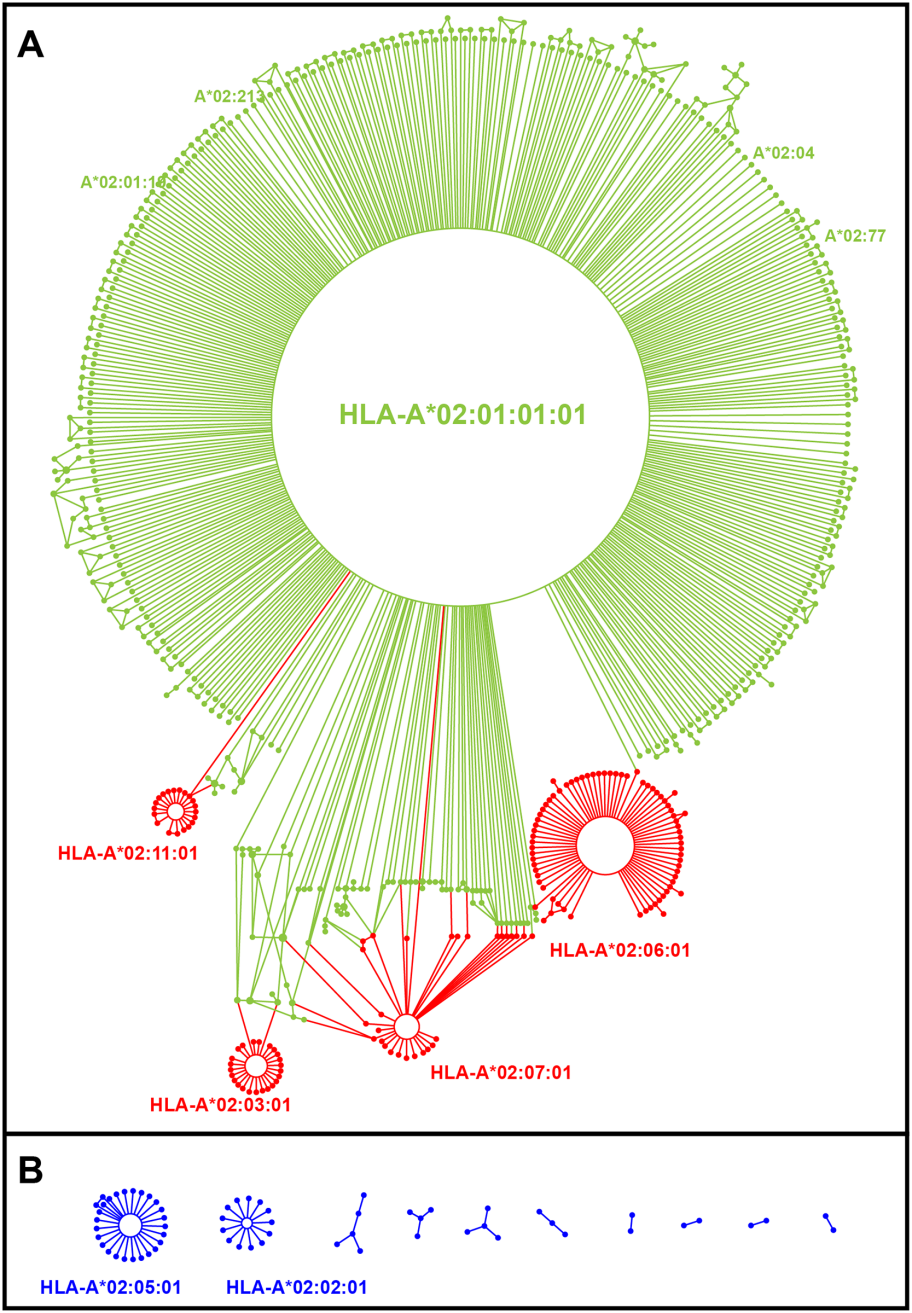
Most alleles within a SEG are related by single point substitutions. Shows all of the members of the final HLA-A*02:*01*:*01*:*01* SEG. **A** The parental HLA-A*02:*01*:*01*:*01* allele has 423 “child” alleles that vary from A*02:*01*:*01*:*01* by a point substitution (green). Additional alleles can be connected by two or more point substitutions (red). In the algorithm the intermediate SEGs, for example HLA-A*02:*07*:*01*, are constructed and subsequently added to other larger SEGs. **B** Ten other HLA-A*02 SEGs were identified that could not directly be linked to the HLA-A*02:*01*:*01*:*01* SEG because they differed from it by more than one point substitution and no intermediate alleles were identified. All of the SEGS with more than a single child are derived by intragenic recombination. Six of the seven are based on an intragenic recombinant involving the HLA-A*02:*01*:*01*:*01* SEG. The seventh is the core allele HLA-A*02:05:01.

For all pairs of alleles, the Sq2 algorithm determines the minimum number of substitutions that is needed to convert one allele to the other. This is achieved by calculating the hamming distance (HamD) a measure developed in the field of information theory [54]. Two alleles with a HamD of 1 differ by a single nucleotide substitution. Sq2 identifies chains, clusters and networks of allele that are connected by steps of one substitution. Each connected set of alleles forms a single event group (SEG), an example being the cluster of 453 HLA-A*02 alleles that each differ from HLA-A*02:*01*:*01*:*01* by one nucleotide substitution (Fig 8).

In pairs of alleles that differ by one nucleotide substitution, one allele (the parent) is ancestral and the other (the child) is derived. Having defined all possible allele pairs with a HamD of 1, the algorithm initially gives equivalent consideration that each member of the pair is the parent. The number of child alleles for each candidate parent is usually very different, so the algorithm then assigns the allele with the most children as the parent. For the A*02:*01*:*01*:*01* SEG it is obvious, because A*02:*01*:*01*:*01* has 423 potential children, very few of which have any potential children apart from A*02:*01*:*01*:*01*. Assignment of the parent can lead to additional alleles being incorporated into the SEG. Initially, A*02:*35*:*01* was assigned its own SEG, because it differs from HLA-A*02:*01*:*01*:*01* by two nucleotide substitutions and has 5 child alleles. There are two child alleles that are a single SNP from both the HLA-A*02:*11*:*01* and HLA-A*02:*01*:*01*:*01 SEGs*. The HLA-A*02:*35*:*01* group can be absorbed into either of these larger groups and in this case HLA-A*02:*01*:*01*:*01* SEG is used, as it contains the most children. The analysis also revealed closed circular connections between several alleles, a consequence of different mutations occurring at the same nucleotide position (Fig 8).

A large majority of *HLA-A*, -*B* and -*C* alleles are SNP alleles. To identify recombinant and core alleles, the SNP alleles were first removed from the sequence alignments.

#### Identification of recombinant alleles and core alleles—*sq2_v3*.*pl*

The RDP2 [55] and TOPALi [56, 57] methods that are much used for identifying recombination events are based on mathematical models [58, 59] that assume recombination is infrequent. For *HLA class I* alleles that is not the case, particularly for our reduced dataset that is enriched for recombinant alleles. In testing the capacity of RDP2 and TOPALi to identify recombinant *HLA class I* alleles, we found these programs only identified a subset of the recombinants, and these were ones that were obvious from visual inspection.

To improve the detection of recombinants within the reduced *HLA class I* dataset we developed another algorithm that relies on pattern matching. This part of Sq2 uses HamD to identify the most similar alleles, and the motifs that distinguish them. The algorithm treats the gain or loss of sequence motifs as single events, and generates Motif Event Groups (MEGs) based upon the analysis of these events. For example, exchange of the Bw4 for the Bw6 motif [28, 60] at nucleotide positions 310–320 in *HLA-B* is considered a single event. *HLA-B* alleles differing only by Bw4/Bw6 are placed in the same MEG. The output of the analysis is a series of MEGs, for which the size and content is compared and parent alleles selected. These MEG parents are candidate core alleles.

This methodology is repeated in an iterative manner, comparing alleles in the data set and the motifs generated. This permitted further reduction of the database as two or more alleles were assigned to a MEG. Where possible this was done programmatically, providing an initial reduced set of alleles. Visual inspection was then performed to facilitate identification of more complicated patterns of recombination. To aid this analysis, invariant positions in the sequence were removed from alignments. The alignments were based on the consensus sequence and the alleles ordered by their increasing deviation from the consensus. No alleles having a unique nucleotide substitution were removed from the dataset. For some MEGs, determination of the core allele could not be achieved programmatically. In those cases, the global distributions of the candidate alleles were compared and the most widely distributed allele chosen as the MEG progenitor. After removing SNP and recombinant alleles, the database was reduced to a set of 42 core alleles: 11 *HLA-A*, 17 *HLA-B* and 14 *HLA-C*.

Identification of core alleles enabled us to refine the characterization, definition and assignment of recombinant motifs using a computational algorithm. Starting at the 5′end of exon 2, each core allele was compared to the allele under study for identity at each nucleotide position. When a difference was detected the comparison was stopped. For all core alleles, these regions of identity were compared and the longest one selected. The process was then started again from the position following the 3′end of the first identity region, and was continued through to the 3′end of exon 3. This produced a profile of regions with identity to the core alleles. The process was then repeated, but was started at the 3′end of exon 3 and then proceeded to the 5′end of exon 2. The two profiles were then combined to produce a map of potential recombinant regions, which highlighted the maximum and minimum areas needed for recombination. The algorithm was also seeded with both core alleles and known recombinants, which allowed the identification of second or third generation recombinants. The algorithm was also used to identify intergenic and intragenic recombination events.

### Targeted dN-dS analysis of positive selection

The MEGA software package, version 5.1, [61–63] was used to assess positive selection in *HLA-A -B*, and -*C* sequences using the codon-based *Z*-test of selection. The analysis used the Kumar method [64], with variance of the difference being computed by the bootstrap method (1,000 replicates, to allow for multiple testing). For each gene dN and dS analysis examined the exon 2 and 3 sequences, both separately and combined. Further to this, analysis was performed on specific codons. In these experiments, the nucleotide positions of interest were extracted from each allele and an artificial sequence created. All allele sequences were then compiled into a single data file that could be run through the dN-dS analysis. Each of these data sets was analysed for the positions across the combined exon 2 and 3 sequence, as well as for the positions within the individual exons. In all cases, the probability of rejecting the null hypothesis of strict-neutrality (dN = dS) in favor of the alternative hypothesis (dN>dS) is shown in Tables 2 and 4. P-values were subject to Bonferroni correction for multiple comparison. P-values of less than 0.05 are considered significant at the 5% level and are highlighted.

### Phylogenetic tree construction

Full-length coding sequences of the core alleles were obtained from the IPD-IMGT/HLA Database. Sequences were aligned in Geneious 7 [65] using the MAFFT algorithm [66]. The alignment was input into MEGA 7 and the tree was constructed using the Neighbor Joining method with pairwise deletion, the Tamura-Nei model, and 1000 bootstrap replicates. It is displayed as an unrooted tree in Fig 6B [67, 68] and bootstrap values of >50 are shown on the tree.

### Maps of allele frequency in human populations

For the core alleles, maps of their frequency distribution in human populations were generated using ArcGis 10 [69]. Population allele frequencies and location coordinates were downloaded from allelefrequencies.net [70]. Only anthropologically well-characterized populations of >50 individuals were included. Specifically excluded were admixed populations, populations of recent migrants, bone marrow registry populations and the subjects of disease association studies. Populations with low resolution HLA class I typing, (less than two field, four digit resolution) were not included in the final dataset.

## Supporting information

S1 Fig The rate of data acquisition increased with improvements in clinical HLA typing. Shown are the numbers of HLA-A [blue], HLA-B [red], and HLA-C [green] sequences deposited in the IPD-IMGT/HLA Database at various times between 1988 and 2016. The slope of each curve gives the rate of data acquisition. The colored bars just above the x-axis denote time periods when different methods of clinical HLA typing were dominant. Green; a period when serological HLA typing dominated and techniques to clone and sequence HLA alleles were first developed. This gave sequences for the common alleles. Purple; DNA based methods for typing HLA class I alleles were developed and applied. Most of these techniques were based upon sets of oligonucleotide probes. These methods had limited potential to detect ‘new’ alleles. Grey; the period in which high resolution probe-based typing was developed and first applied to typing the large panels of donors needed for bone marrow transplantation. These methods had improved potential to detect ‘new’ alleles Orange; high resolution high-volume sequence-based typing is introduced and begins to be applied to the millions of donors in the bone marrow transplant registries. These methods detect every ‘new’ allele.
(TIF)

S2 Fig Nucleotide diversity plots. The plots show the number of different nucleotides (A, C, G, T, indel) seen at each position. Numbering of positions is per the full-length mRNA sequence. Bars extending below the baseline indicate conserved positions with only a single nucleotide present in all alleles.
(TIF)

S3 Fig Most positions in α_1_ and α_2_ have more than one possible nucleotide. Each position can have a maximum of five different nucleotides with the fifth nucleotide being an insertion or deletion. This shows the number of positions in each category.
(TIF)

S4 Fig Amino acid diversity plots. The plots show the number of amino acid residues found at each position. The numbering is the position in the mature protein. Similar to S2 Fig, conserved residues would be shown as bars extending below the baseline. There were no conserved residues in any of the genes (Table 1).
(TIF)

S5 Fig Polymophic positions: Where the second nucleotide has a frequency >1%. Shown for *HLA-A*, *HLA-B* and *HLA-C* are all positions in exons 2 and 3 where the second most common nucleotide has a frequency >1%. The table shows the frequency of the first and second most common nucleotides. The final column shows all of the nucleotides seen at the position in descending order of frequency.
(TIF)

S6 Fig Polymorphic positions: Where second amino acid has a frequency >1%. Shown for HLA-A, HLA-B and HLA-C are all positions in the sequence of the α_1_ and α_2_ domains where the second most common amino acid has a frequency >1%. The table shows the frequency of the first and second most common amino acids. Highlighted yellow are residues that contribute to the antigen recognition site.
(TIF)

S7 Fig Maps showing the distributions of HLA core alleles in human populations. Allele frequencies and location coordinates were downloaded from allelefequencies.net. Only populations of greater than 50 individuals, and for which samples were collected for anthropologic studies, were included in the analysis.
(PDF)

S8 Fig Pairwise comparison of the nucleotide sequences of HLA class I core alleles. The dot plots show the results of pairwise comparison of the nucleotide sequences of the core alleles (Fig 6). A color scale indicates the number of nucleotide differences in each pair compared with red representing the most closely related alleles. The grid shows the values for the pair-wise differences within each gene for the complete set of alleles and the core alleles.
(TIF)

S9 Fig Statistics for all pairwise differences. The upper panel shows the values for the pairwise distance calculations displayed in Fig 7. Entries are the numbers of calculations performed. Values are shown for all gene calculations indicated by A, B, or C, intra-group (within SEGs), inter-group (between SEGs), and cores. In addition, comparison between pairs of genes, all intra-gene and all inter-gene is also shown. The lower panel provides the values for the distance of individual comparison from the mean value for each of the groupings. For example, in the line for the HLA-A gene (A) the minimum distance from the mean value is 0.18, representing 23 differences, the maximum distance is 34.18, representing the maximum value of 57 differences, and the remaining values describe the average distances from the mean.
(TIF)
