# Supplementary figures and images for: Distinguishing functional polymorphism from random variation in the sequences of >10,000 HLA-A, -B and -C alleles
Source: PLoS Genet. 2017 Jun 26;13(6):e1006862. doi: 10.1371/journal.pgen.1006862 (PMC5507469; doi:10.1371/journal.pgen.1006862)

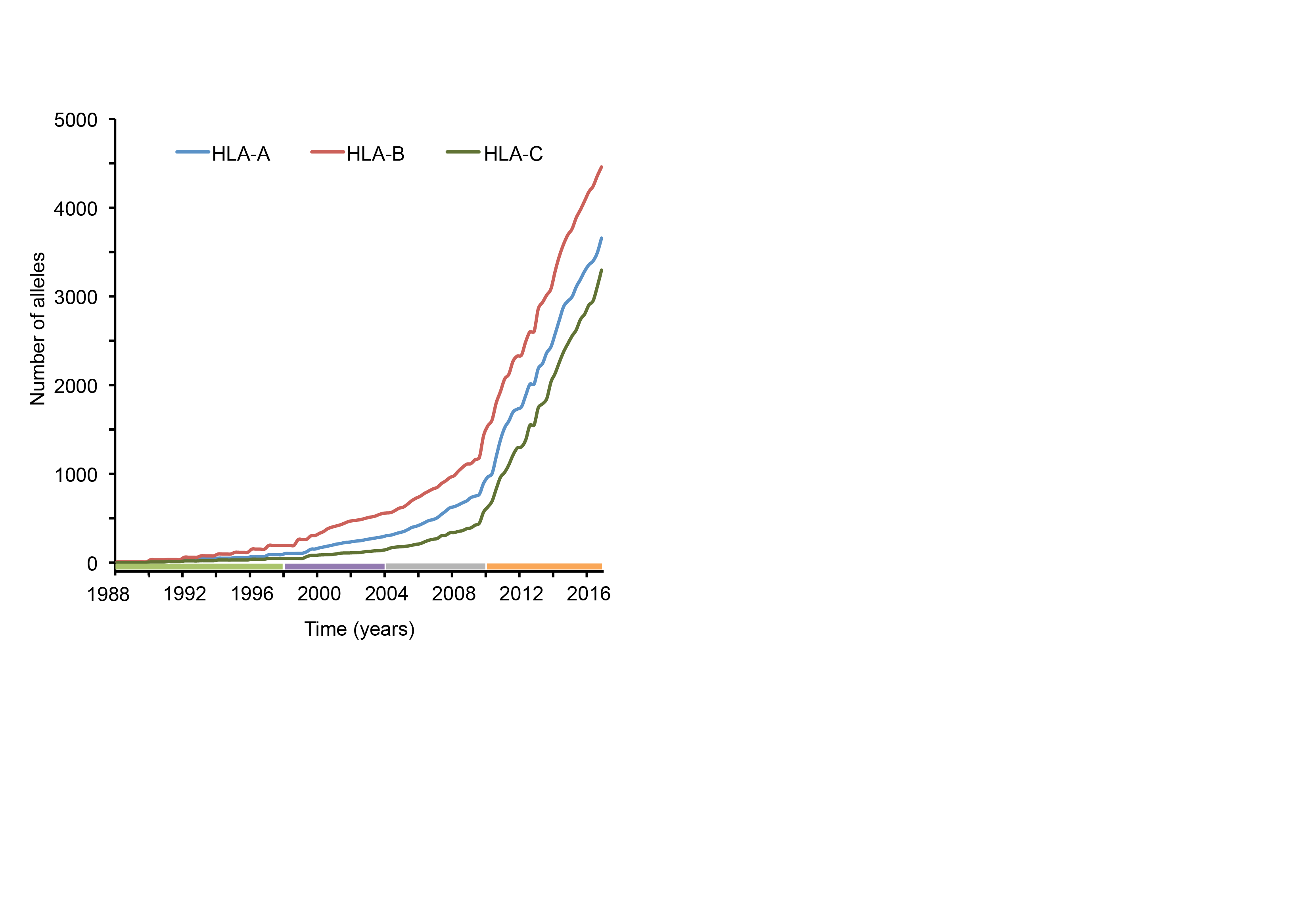

Supplement: S1 Fig — Shown are the numbers of HLA-A [blue], HLA-B [red], and HLA-C [green] sequences deposited in the IPD-IMGT/HLA Database at various times between 1988 and 2016. The slope of each curve gives the rate of data acquisition. The colored bars just above the x-axis denote time periods when different methods of clinical HLA typing were dominant. Green; a period when serological HLA typing dominated and techniques to clone and sequence HLA alleles were first developed. This gave sequences for the common alleles. Purple; DNA based methods for typing HLA class I alleles were developed and applied. Most of these techniques were based upon sets of oligonucleotide probes. These methods had limited potential to detect ‘new’ alleles. Grey; the period in which high resolution probe-based typing was developed and first applied to typing the large panels of donors needed for bone marrow transplantation. These methods had improved potential to detect ‘new’ alleles Orange; high resolution high-volume sequence-based typing is introduced and begins to be applied to the millions of donors in the bone marrow transplant registries. These methods detect every ‘new’ allele. (TIF) [file pgen.1006862.s001.tif]

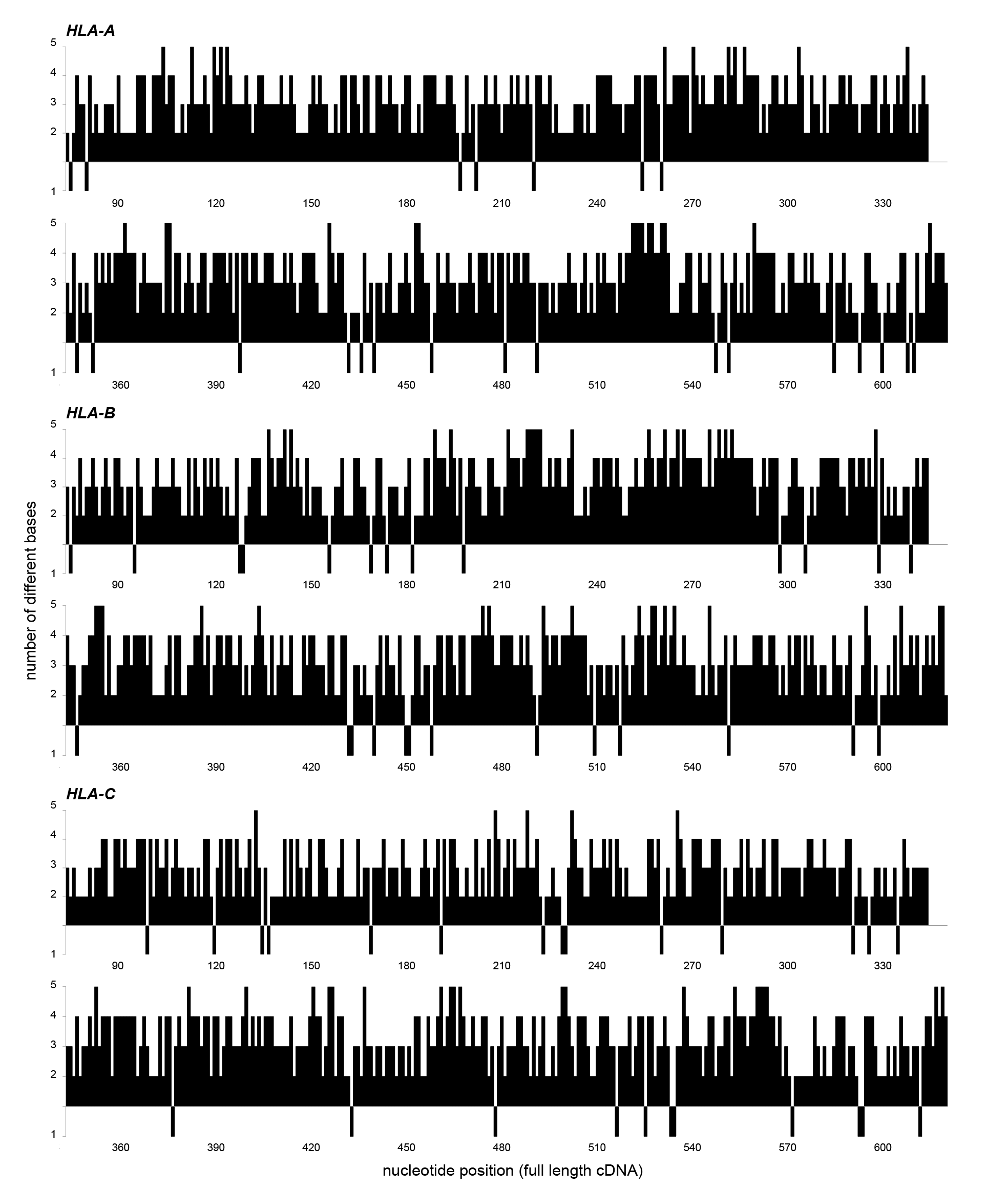

Supplement: S2 Fig — The plots show the number of different nucleotides (A, C, G, T, indel) seen at each position. Numbering of positions is per the full-length mRNA sequence. Bars extending below the baseline indicate conserved positions with only a single nucleotide present in all alleles. (TIF) [file pgen.1006862.s002.tif]

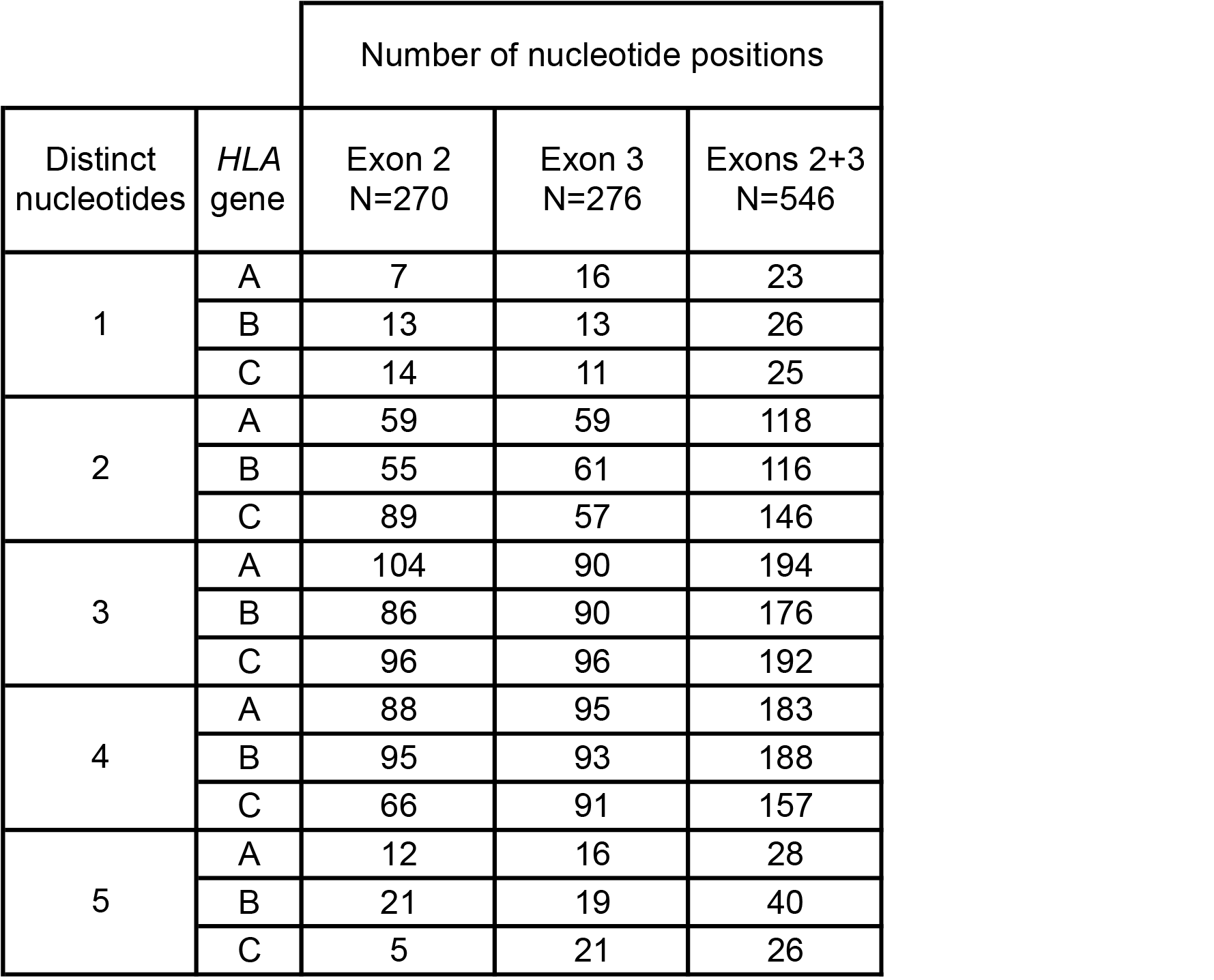

Supplement: S3 Fig — Each position can have a maximum of five different nucleotides with the fifth nucleotide being an insertion or deletion. This shows the number of positions in each category. (TIF) [file pgen.1006862.s003.tif]

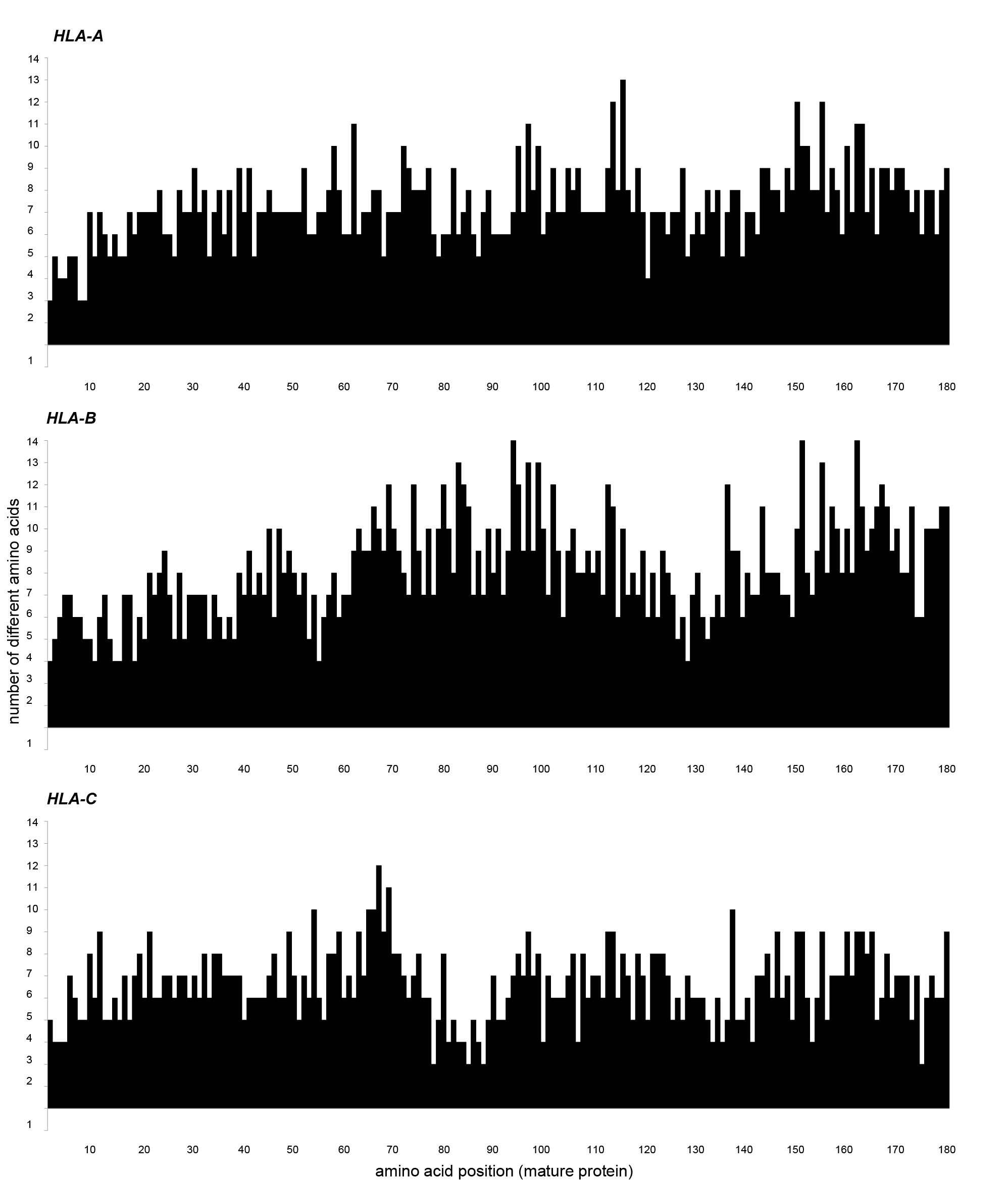

Supplement: S4 Fig — The plots show the number of amino acid residues found at each position. The numbering is the position in the mature protein. Similar to S2 Fig, conserved residues would be shown as bars extending below the baseline. There were no conserved residues in any of the genes (Table 1). (TIF) [file pgen.1006862.s004.tif]

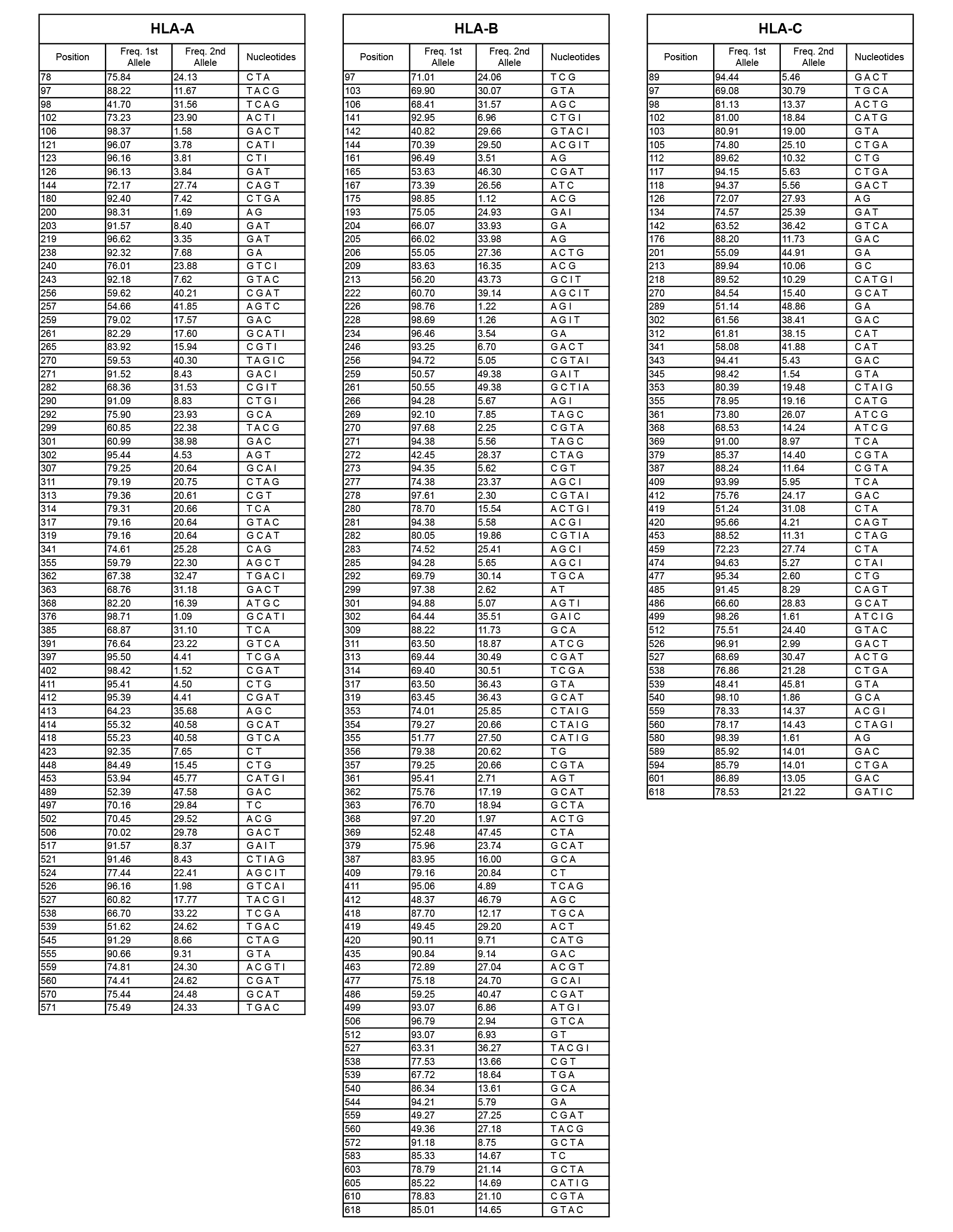

Supplement: S5 Fig — Shown for HLA-A, HLA-B and HLA-C are all positions in exons 2 and 3 where the second most common nucleotide has a frequency >1%. The table shows the frequency of the first and second most common nucleotides. The final column shows all of the nucleotides seen at the position in descending order of frequency. (TIF) [file pgen.1006862.s005.tif]

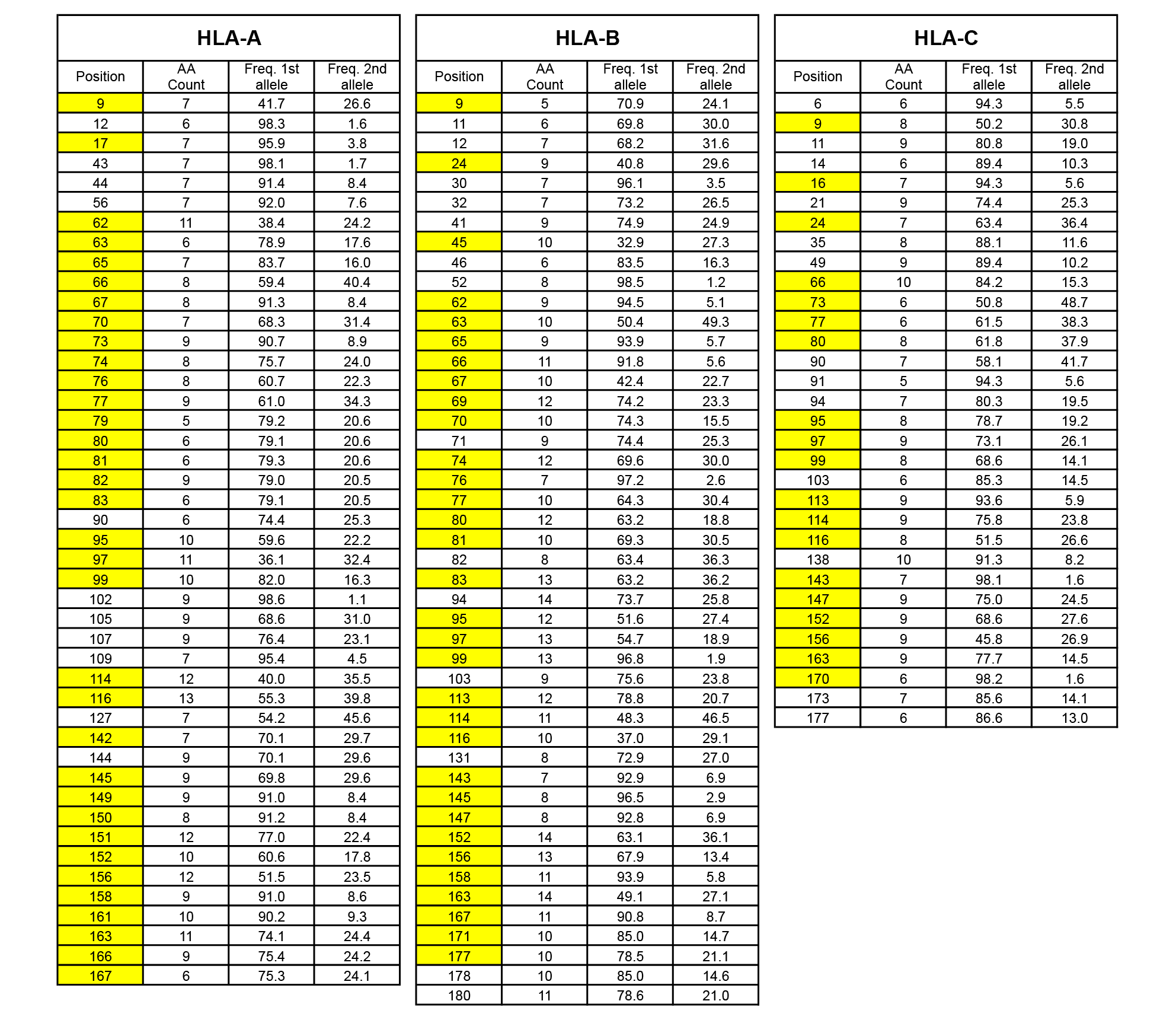

Supplement: S6 Fig — Shown for HLA-A, HLA-B and HLA-C are all positions in the sequence of the α1 and α2 domains where the second most common amino acid has a frequency >1%. The table shows the frequency of the first and second most common amino acids. Highlighted yellow are residues that contribute to the antigen recognition site. (TIF) [file pgen.1006862.s006.tif]

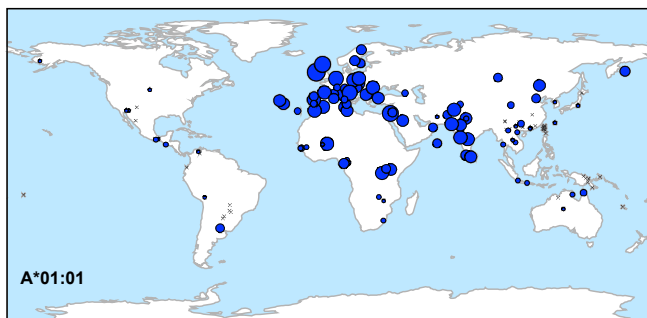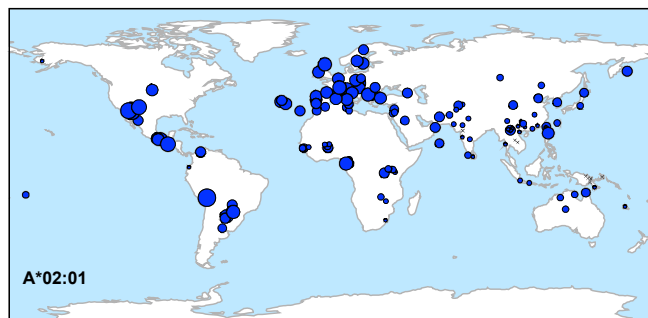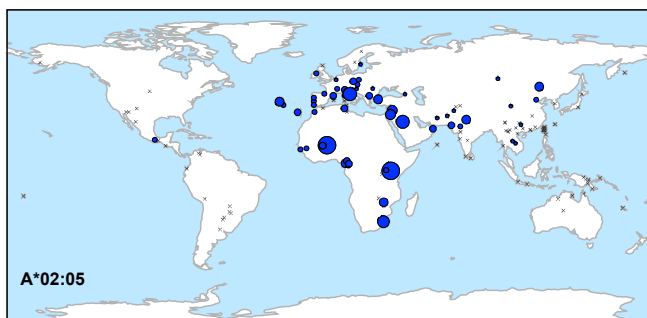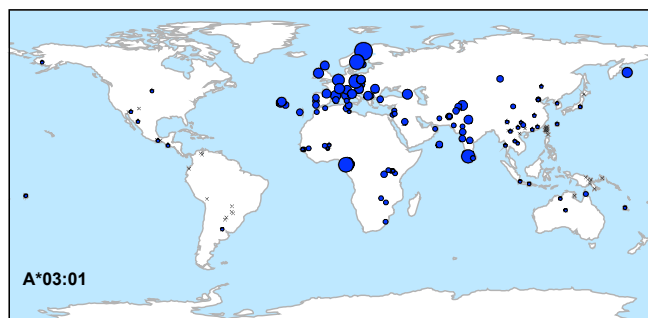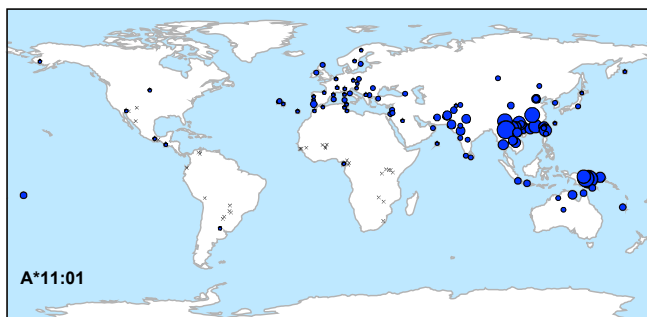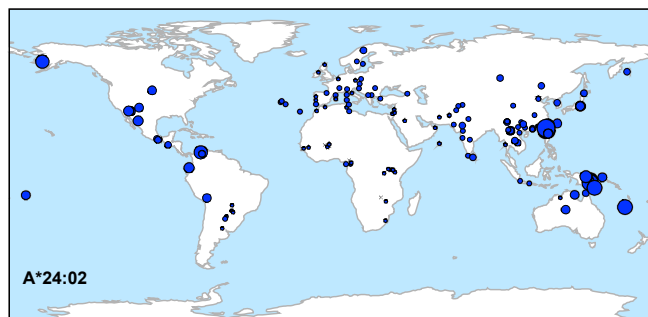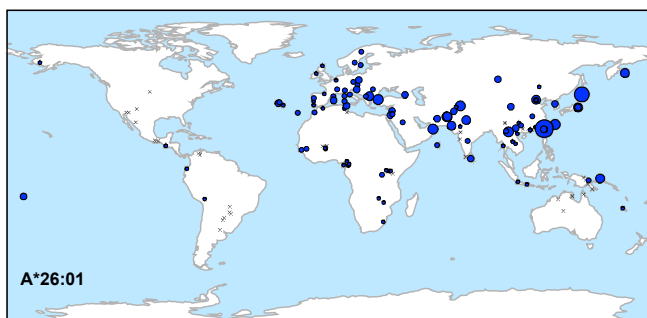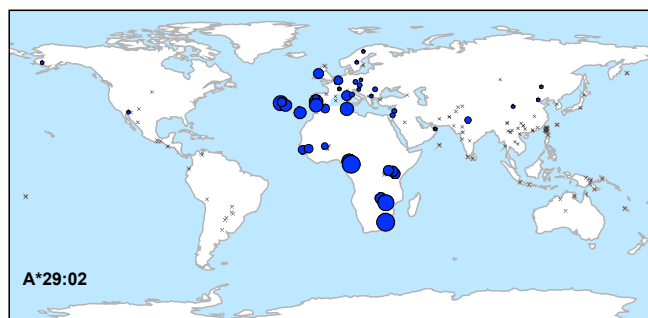

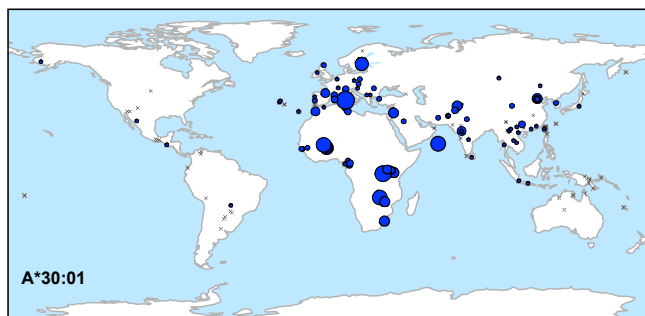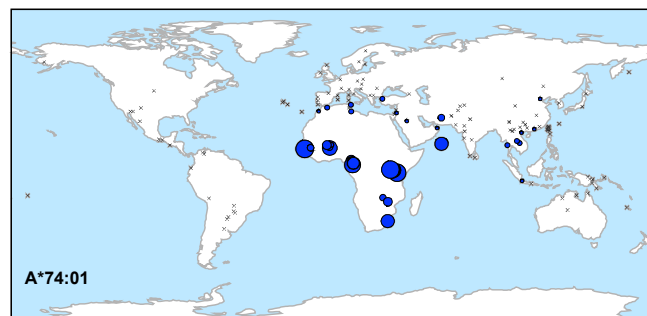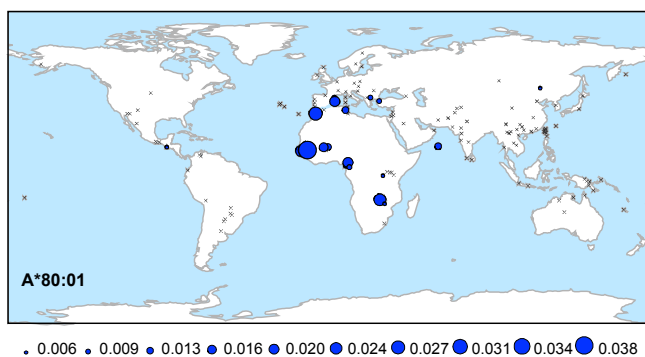

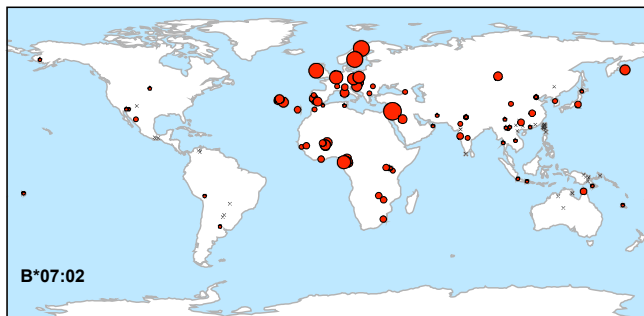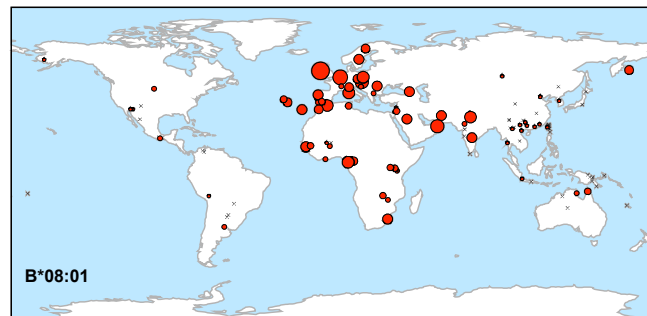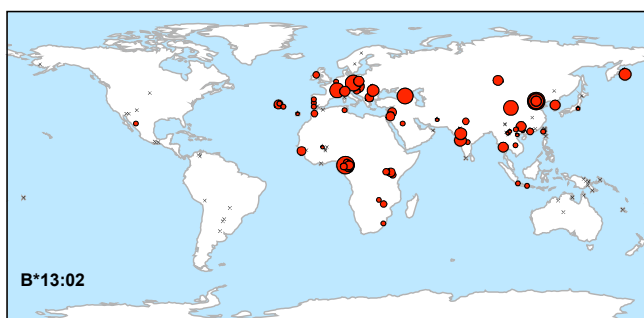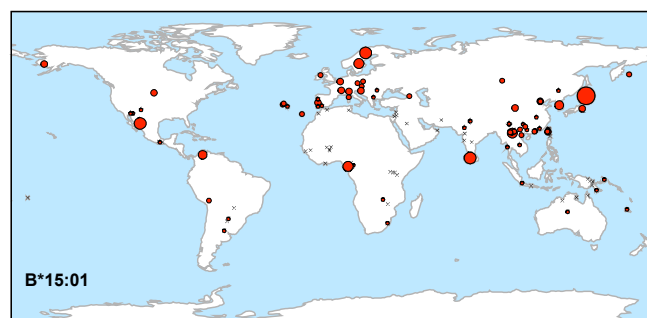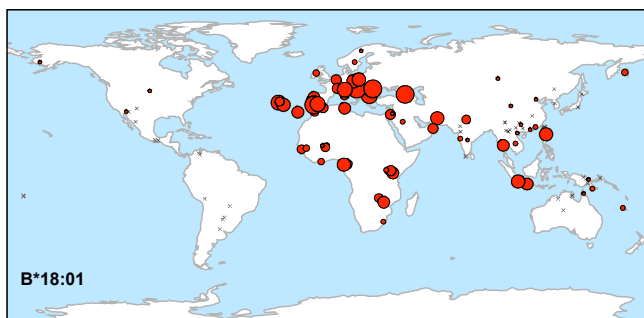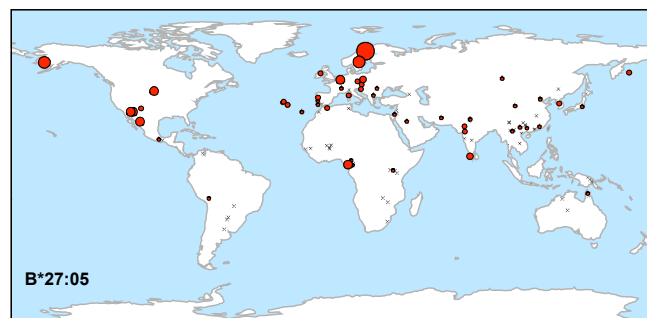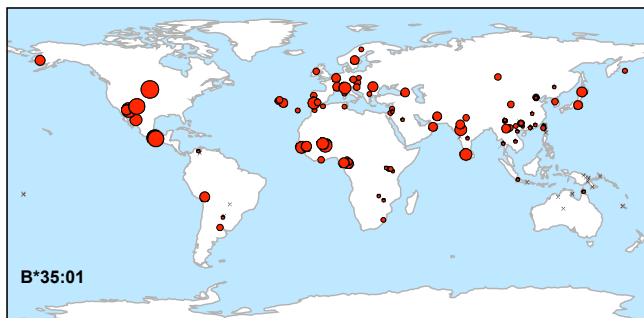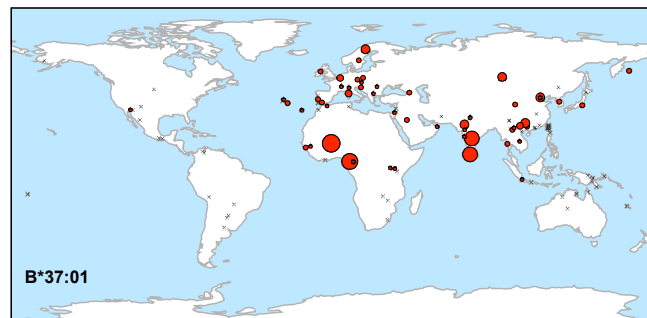

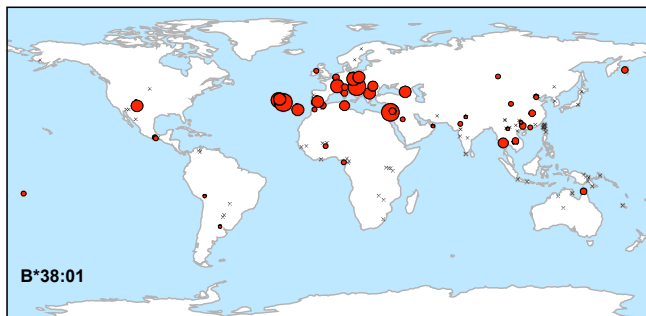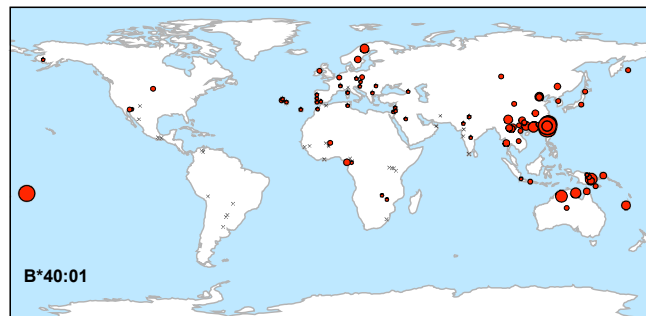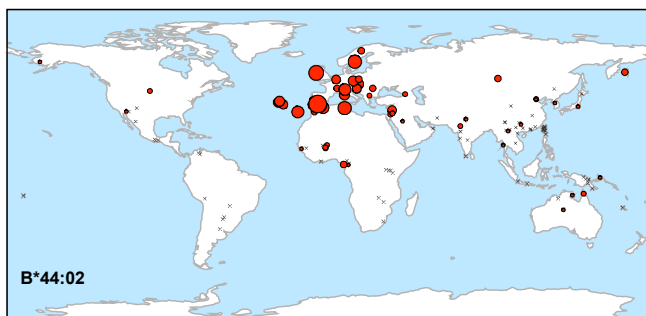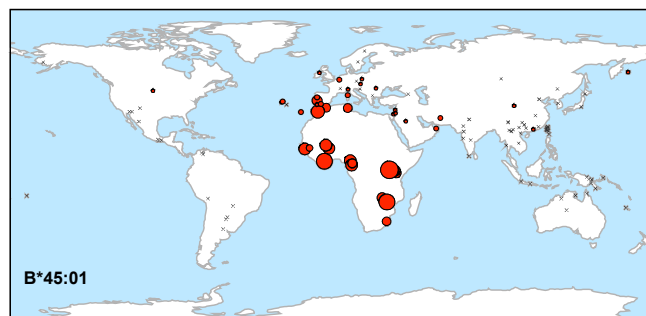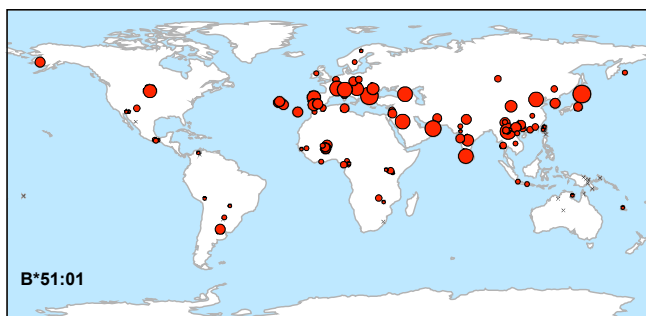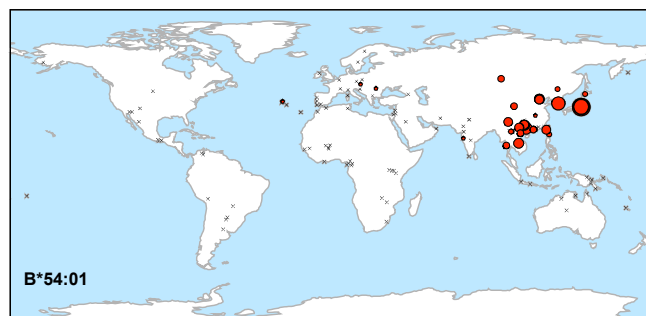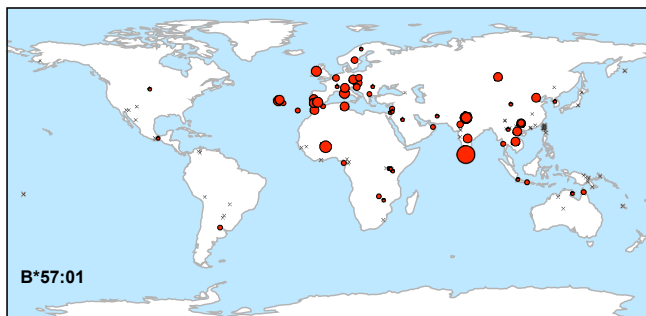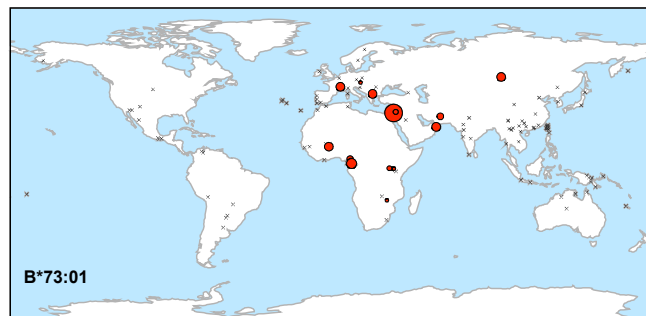

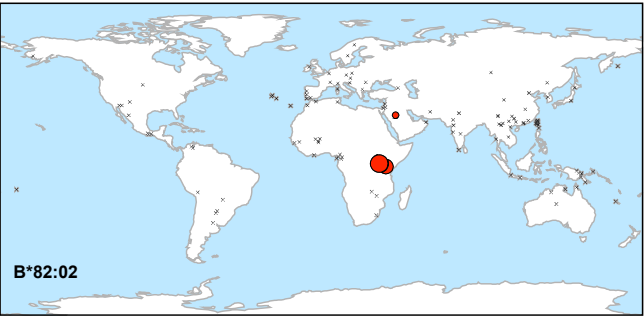

• 0.001 • 0.002 • 0.003 • 0.004 • 0.005 • 0.006 • 0.007 • 0.008 • 0.009 • 0.010

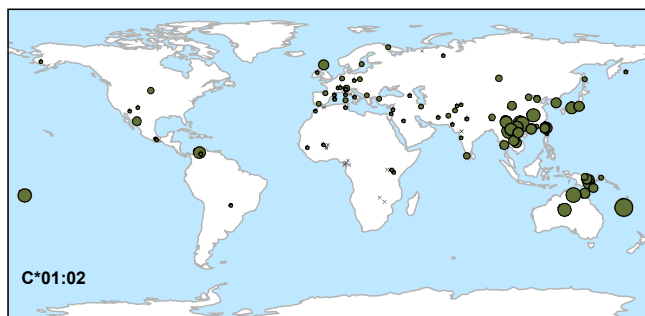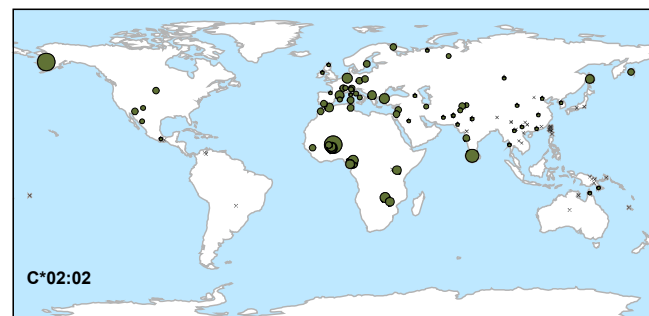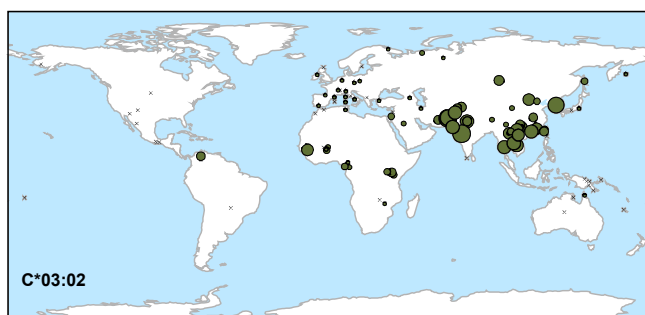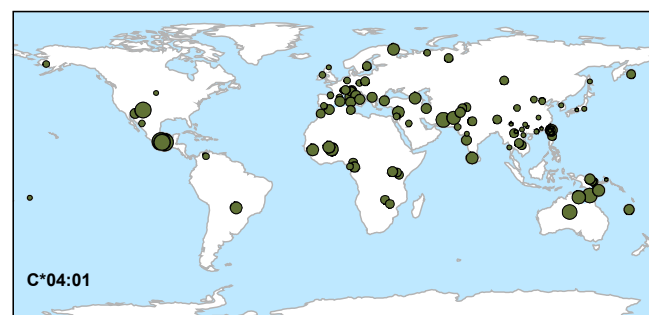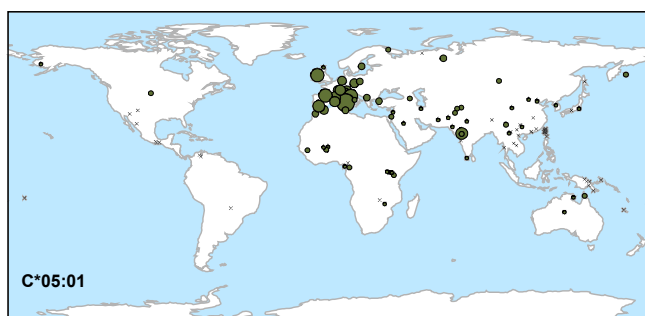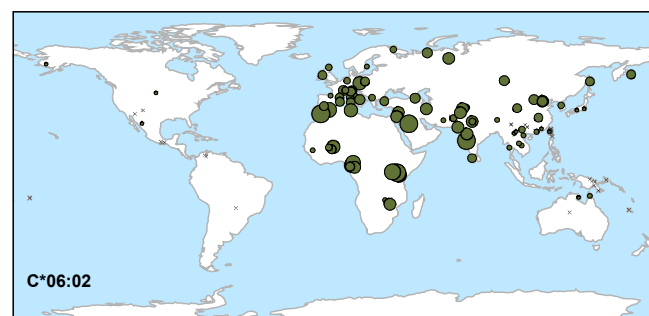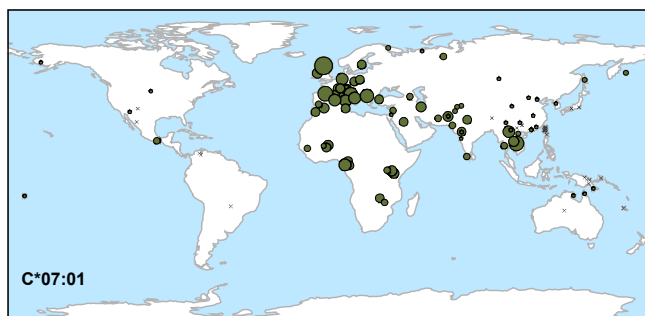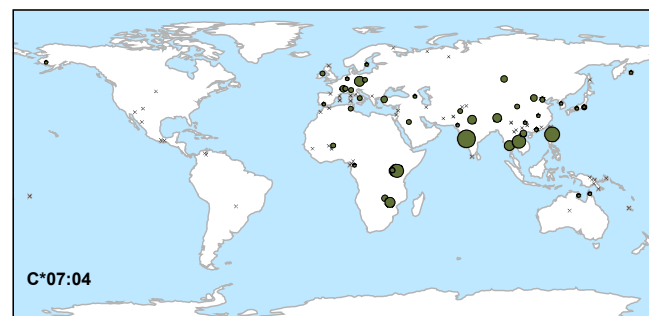

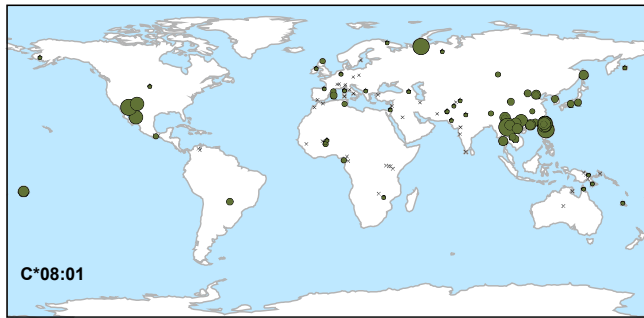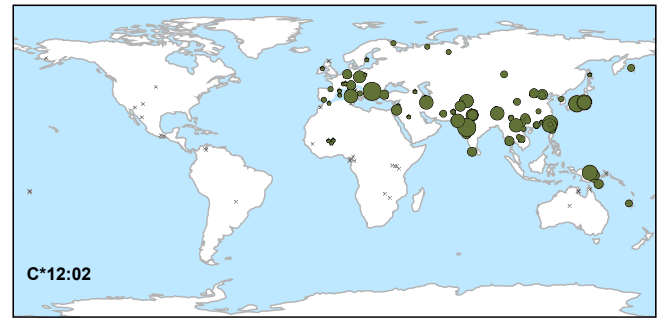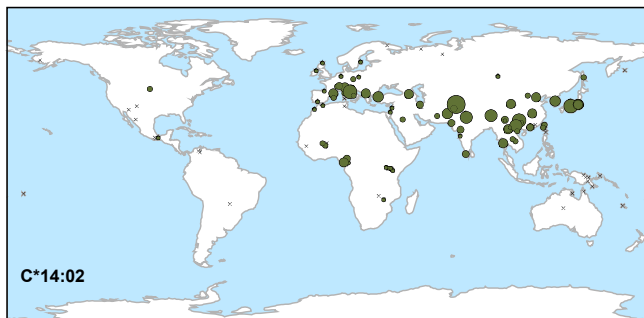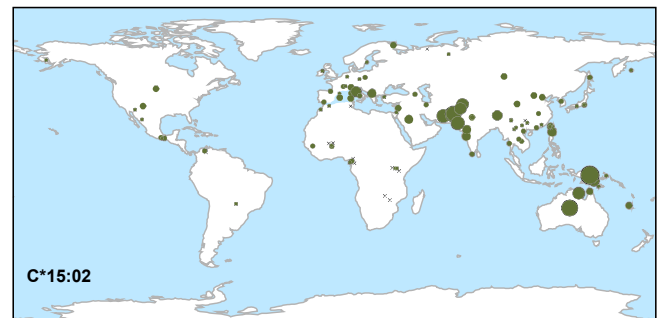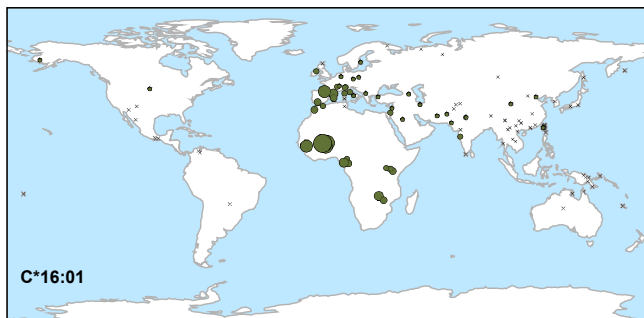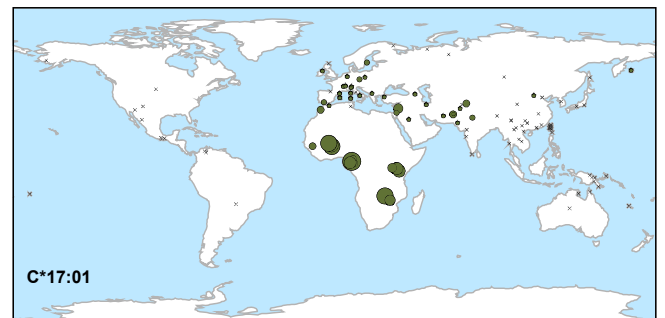

Supplement: S7 Fig — Allele frequencies and location coordinates were downloaded from allelefequencies.net. Only populations of greater than 50 individuals, and for which samples were collected for anthropologic studies, were included in the analysis. (PDF) [file pgen.1006862.s007.pdf]

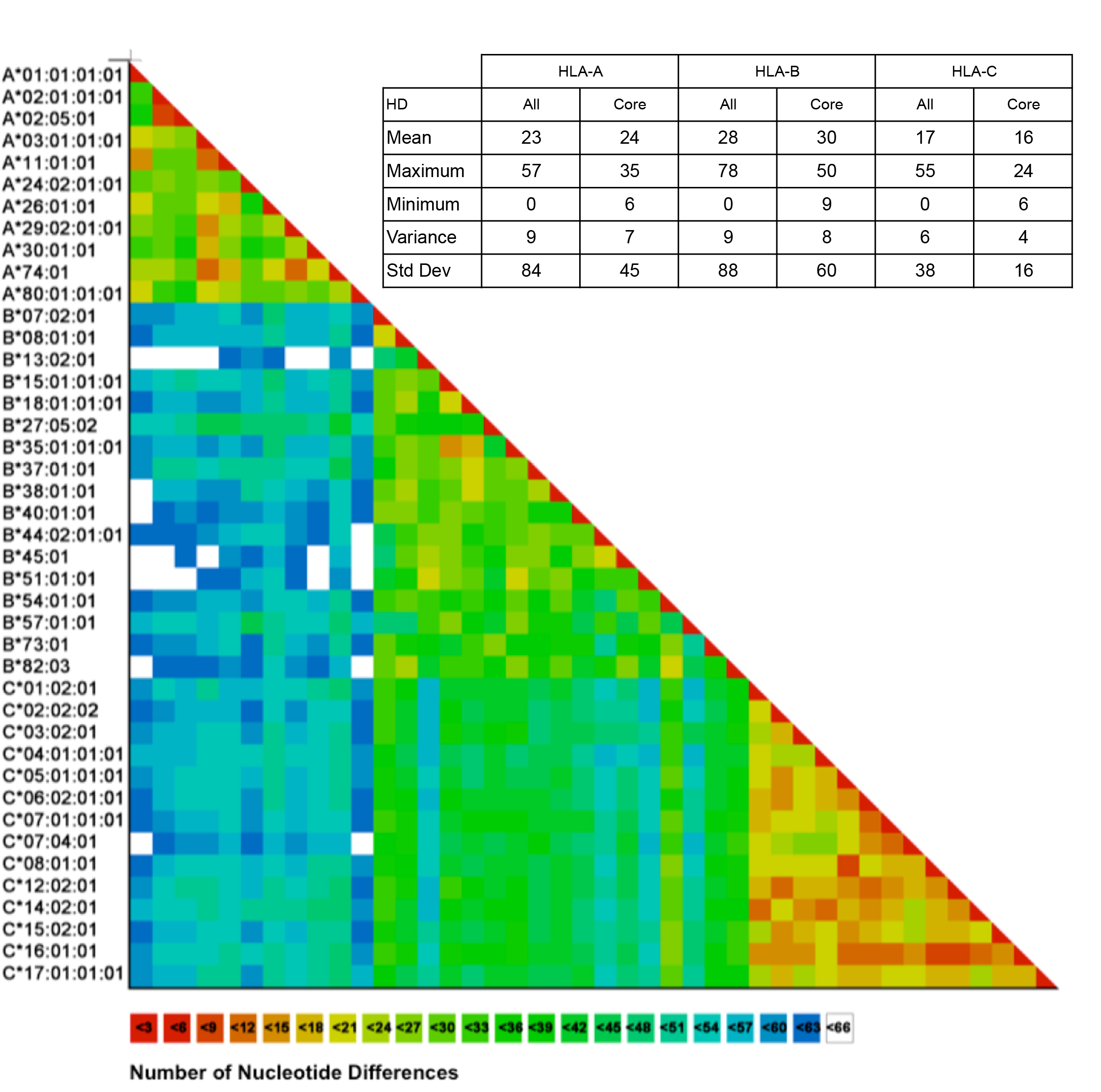

Supplement: S8 Fig — The dot plots show the results of pairwise comparison of the nucleotide sequences of the core alleles (Fig 6). A color scale indicates the number of nucleotide differences in each pair compared with red representing the most closely related alleles. The grid shows the values for the pair-wise differences within each gene for the complete set of alleles and the core alleles. (TIF) [file pgen.1006862.s008.tif]

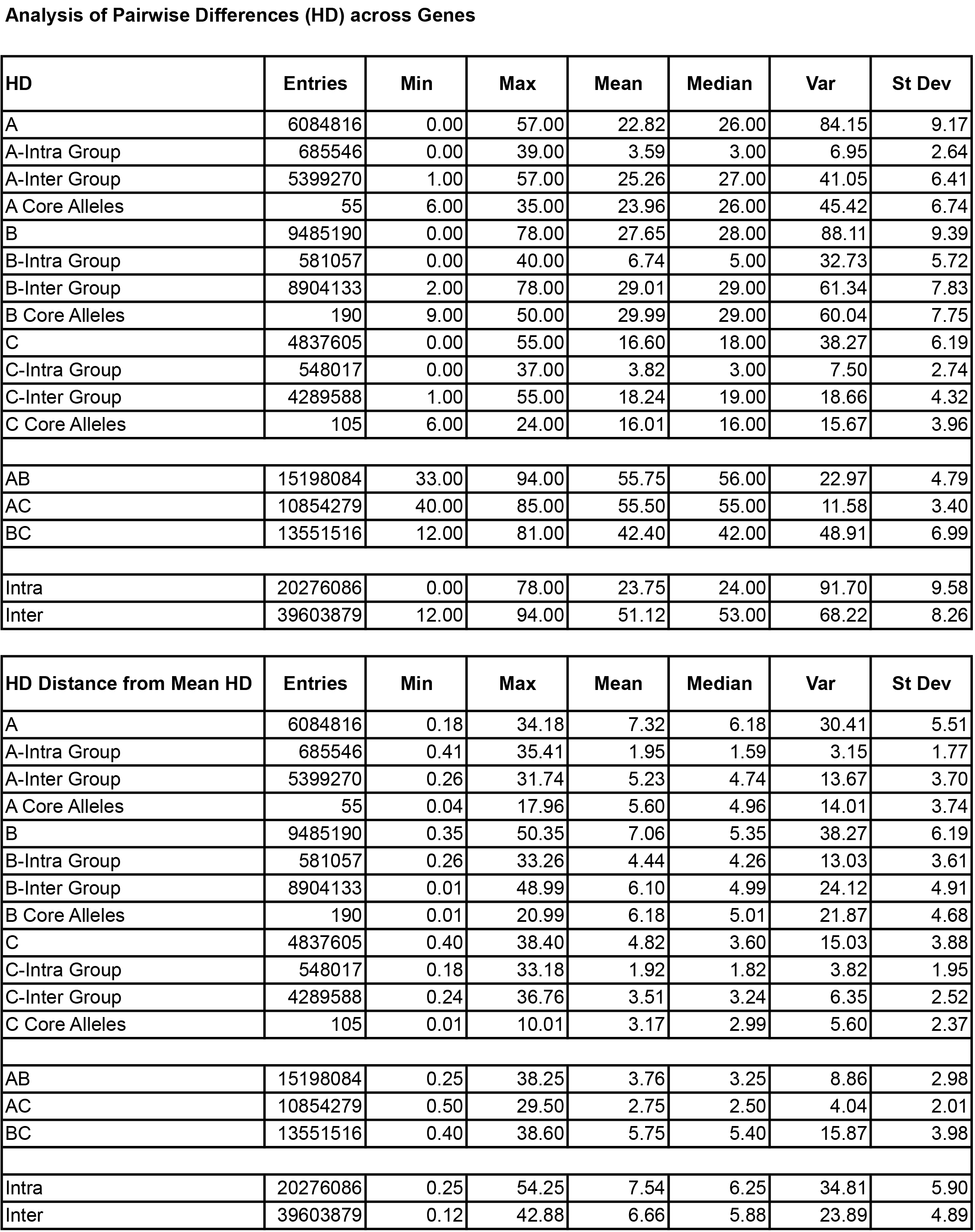

Supplement: S9 Fig — The upper panel shows the values for the pairwise distance calculations displayed in Fig 7. Entries are the numbers of calculations performed. Values are shown for all gene calculations indicated by A, B, or C, intra-group (within SEGs), inter-group (between SEGs), and cores. In addition, comparison between pairs of genes, all intra-gene and all inter-gene is also shown. The lower panel provides the values for the distance of individual comparison from the mean value for each of the groupings. For example, in the line for the HLA-A gene (A) the minimum distance from the mean value is 0.18, representing 23 differences, the maximum distance is 34.18, representing the maximum value of 57 differences, and the remaining values describe the average distances from the mean. (TIF) [file pgen.1006862.s009.tif]
